# QuickConc Vacuum: A high-sensitive cationic-assisted method for large-volume eDNA concentration and sensitive biodiversity monitoring^☆^

**DOI:** 10.1016/j.mex.2026.104126

**Published:** 2026-08-27

**Authors:** Tomohiro Kuroita, Qianqian Wu, Ryo Iwamoto, Toshifumi Minamoto

**Affiliations:** a AdvanSentinel Inc., 3-1-8, Doshomachi, Chuo-ku, Osaka, 541-0045, Japan; b Shionogi & Co., Ltd, 3-1-8, Doshomachi, Chuo-ku, Osaka, 541-0045, Japan; c Graduate School of Human Development and Environment, Kobe University, 3-11 Tsurukabuto, Nada-ku, Kobe, Hyogo, 657-8501, Japan

**Keywords:** Environmental DNA, Biodiversity monitoring, Sea water, Filtration, PCR, Metabarcoding

## Abstract

Environmental DNA (eDNA) analysis enables biodiversity surveys with minimal disturbance to aquatic environment. We previously developed QuickConc to capture eDNA by combining dispersed glass fibers with quaternary ammonium surfactants, but manual processing limited small volumes. Here, we introduce QuickConc Vacuum (QCV), an advanced protocol adapting this cationic-assisted technology for vacuum filtration to process liter-scale samples. We performed comparisons with glass fiber filters (GF) and Sterivex, using aquarium water and field seawater. In aquarium experiments, total eDNA yield positively correlated with volume for all methods, with QCV showing significantly higher total eDNA at 2 L. While species-specific yields showed no significant differences among methods, metabarcoding revealed that QCV and Sterivex detected significantly higher fish-species richness than GF. In field seawater trials, QCV consistently recovered higher eDNA yields and detected significantly more fish species at the 2 L volume compared to other methods. QCV successfully overcomes the volume limitations of the original method while maintaining high eDNA yields, providing a robust solution for high-sensitivity eDNA monitoring in aquatic ecosystems.

• QCV is a vacuum-filtration protocol based on the cationic-assisted capture technology using dispersed glass fibers for efficient eDNA binding.
• QCV is designed to process liter-scale environmental water samples.

## Graphical abstract

**Figure:**
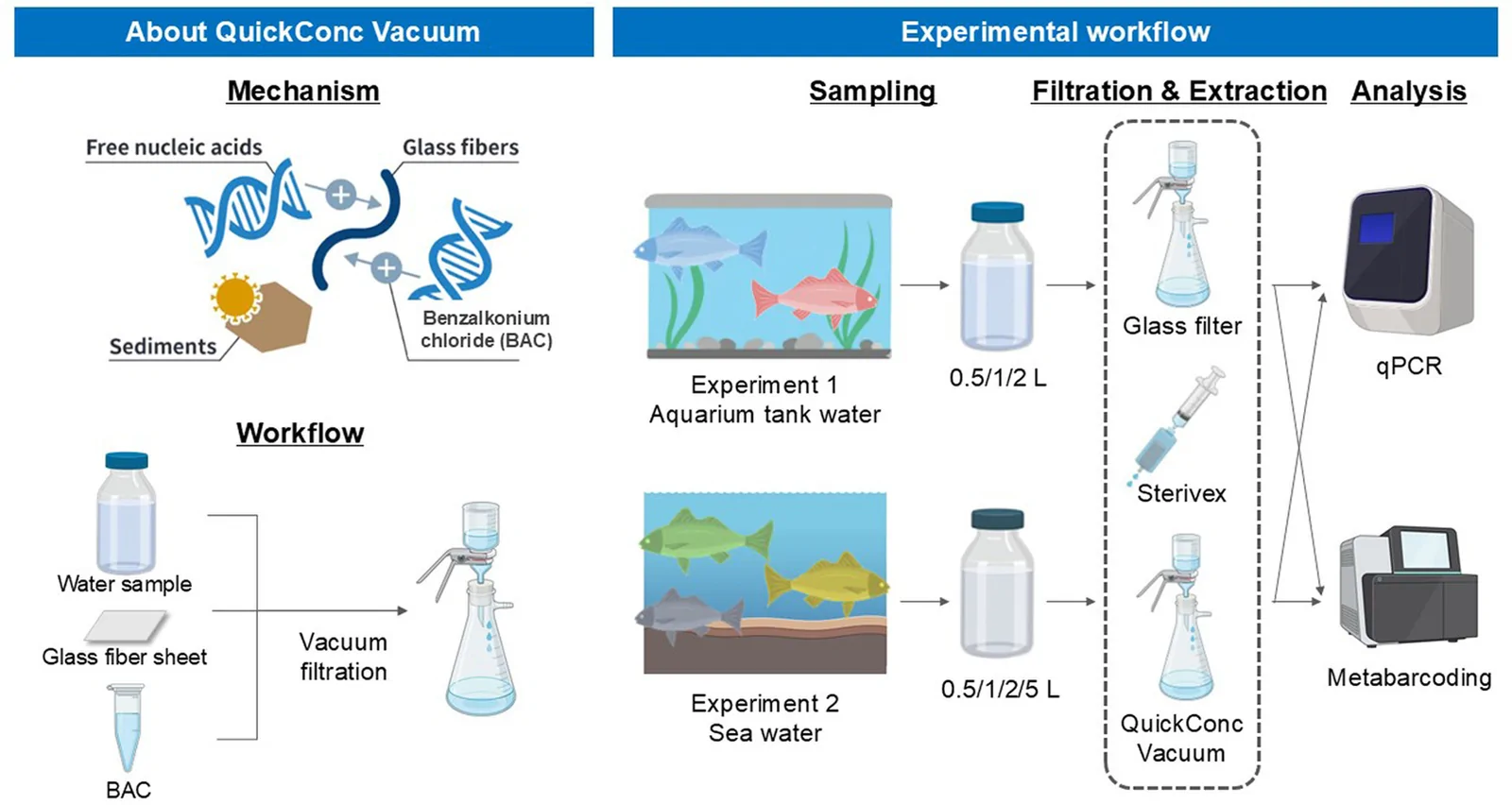

## Background

Specifications table

**Table:** 

**Subject area**	Environmental Science
**More specific subject area**	Environmental DNA
**Name of your method**	QuickConc Vacuum
Name and reference of original method	T. Kuroita, Q. Wu, R. Iwamoto, T. Minamoto, QuickConc: A rapid, efficient, and power‐free eDNA concentration method with cationic‐assisted capture, Ecol. Evol. 15 (2025) e72269. 10.1002/ece3.72269
**Resource availability**	Information on all resources needed to reproduce this procedure is included in the present article.

Environmental DNA (eDNA) approaches support biodiversity surveys by inferring organismal presence from genetic material without disruption of ecosystem [1,2]. The eDNA analysis workflow comprises capture, purification, and reaction [[3], [4], [5]]. The initial capture process is a critical step, as its efficiency is significantly affected by the high variability of environmental water samples such as pH, turbidity, and the types and concentrations of PCR inhibitors. Standard concentration techniques, such as disk or membrane filtration, often face limitations, including clogging in turbid waters, and they are time-consuming [[6], [7], [8]]. We previously introduced QuickConc (QC), a novel concentration strategy that integrates glass fibers with benzalkonium chloride (BAC), a cationic surfactant [9]. This method demonstrated superior eDNA yields compared to widely used filtration methods; however, the original QC protocol was designed as a power-free, manual concentration technique. While advantageous for field portability, the manual approach imposed physical limitations on the volume of water that could be processed. This volume restriction can be a bottleneck when surveying low-density targets in large water bodies, such as oceans, where processing larger sample volumes is essential for maximizing detection probability.

**Fig. 1:**
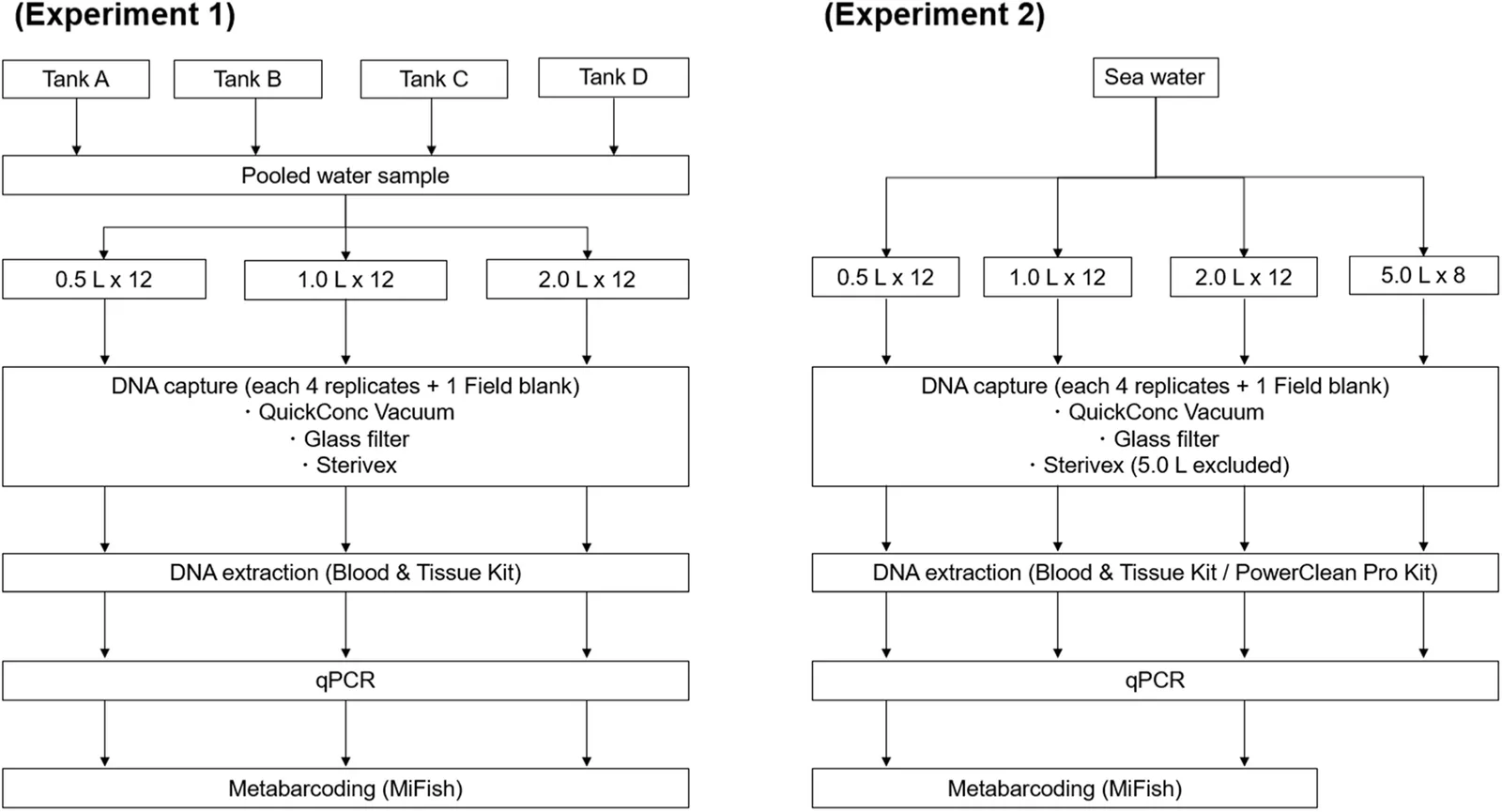
Overview of the experimental workflow. At each volume, four replicates were processed per method in Experiment 1 (aquarium tank water) and Experiment 2 (seawater), and DNA was then extracted. The resulting eDNA extracts were used for metabarcoding and qPCR.

To address the limited throughput of the original manual workflow, we designed QuickConc Vacuum (QCV). This protocol adapts the concept of cationic-assisted capture with glass fibers to a vacuum filtration system. By using vacuum pressure, QCV enables the processing of larger volume samples while retaining the high eDNA yield of the original technology. We assessed performance under both controlled and field-relevant conditions. Experiment 1 used aquarium tank water with a known species composition to assess eDNA yield and species detection in a controlled environment. Experiment 2 applied the method to field seawater samples to evaluate its utility in a natural, large-scale environment. Overview of the experimental workflow is shown in Fig. 1.

## Method details

### Experiment 1: QCV evaluation using aquarium tank water

#### Sample collection of aquarium tank water

Aquarium tank water samples were collected on November 18, 2024, for QCV validation from four tanks (Tanks A, B, C, and D) at Yokohama Hakkeijima Sea Paradise, an aquarium complex in Kanagawa, Japan. They housed 59, 38, 35, and 23 fish species, respectively. These tanks are supplied with natural seawater sourced from the nearby area. Equal volumes from each tank were combined, and the pooled sample was used for the analysis. These tanks were chosen because they had higher species richness than the other tanks in Yokohama Hakkeijima Sea Paradise. Samples were transported to the laboratory under refrigerated conditions immediately after collection. Each method involved collecting four biological replicates along with a single field blank.

#### Filtration and extraction

We evaluated aquarium tank water samples across the following approaches: QCV (AdvanSentinel), glass fiber filter (Advantec), and Sterivex (Millipore). To evaluate the scalability and efficiency of each method, we processed sample volumes of 0.5 L, 1.0 L, and 2.0 L. We added BAC (final 0.001% w/v) and then promptly processed four biological replicates and one field blank with QCV, glass fiber filter (GF), or Sterivex. The detailed protocol for each method is described below.

• **QCV**: The QCV method adapts the dispersed glass fiber technology for vacuum filtration. We applied the previously published workflow for water filtration and eDNA extraction, incorporating slight changes [9]. Briefly, two pieces of glass fiber sheet (1 cm square) and BAC were added to a bottle containing the water sample. We disrupted the glass fiber sheet in the sample to distribute fibers throughout the water phase. The treated water containing the dispersed fibers was then filtered using a standard laboratory vacuum filtration system equipped with the mesh filter to collect the glass fibers. The glass fibers were transferred to a 2 mL tube lysed using a DNeasy Blood & Tissue Kit (Qiagen) workflow with a short proteinase K digestion step (ATL buffer with proteinase K; 56 °C, 30 min). After adding ethanol, we purified the mixture using spin columns according to the manufacturer’s instructions.
• **GF**: We concentrated water samples by vacuum filtration through a glass fiber filter (0.6 µm, GA-55). We performed downstream steps and eDNA extraction using a slightly modified version of the workflow described in the eDNA Society’s sampling and experiment manual [10]. We inserted the filter into a Salivette tube lysed using a DNeasy Blood & Tissue Kit’s workflow with a short proteinase K digestion step (400 μL of ATL buffer with 40 μL proteinase K; 56 °C, 30 min). Following the centrifugation (3000 *g*, 3 min) and addition of 220 μL of nuclease-free water, we mixed the solution with 400 μL of pure ethanol and then purified it on a spin column according to the manufacturer’s instructions.
• **Sterivex**: We used the Sterivex (0.45 µm, SVHV010RS) extraction method. Lysis reagents (Buffer ATL, PBS, Buffer AL, and proteinase K) were introduced into the Sterivex units at the volumes established in previous studies, followed by a 56 °C incubation [11]. After incubation, lysates were processed on spin columns and purified.

We eluted DNA in 100 µL AE buffer and measured DNA yields with using a absorbance spectrophotometry (Nanodrop-1000).

#### qPCR reaction

We quantified *Mugil cephalus* eDNA in aquarium tank water by species-specific qPCR to assess how concentration methods affected eDNA yields. This species was selected because it is highly abundant in the aquarium’s tanks. Based on early studies [12], we selected established primer and probe designs to support specific and dependable amplification (Table S1A). Full reagent conditions are provided in Supplementary Table S1B. Briefly, we quantified target eDNA using a TaqMan-based assay in 20-µL reactions containing Environmental Master Mix 2.0, primers/probe, and 2 µL of template DNA. The qPCR program consisted of a 2-min step at 50 °C and hot-start activation (95 °C, 10 min), followed by a 55-cycle amplification (95 °C for 15 s; 60 °C for 1 min). Standard curves were generated from serially diluted DNA standards (10 to 100,000 copies per reaction). To minimize inter-plate variation, we placed standards, samples, field blanks, and no-template controls on the same qPCR plate and ran each in triplicate. Field blanks and no-template controls were considered negative when none of the replicate reactions showed an exponential amplification. We report standard-curve performance (efficiency and R^2^) in Table S2.

#### Metabarcoding analysis for experiment 1

Metabarcoding analyses were performed to assess how various concentration methods influence the species richness and the composition of the community. The construction of the library commenced with a two-step tailed PCR technique utilizing four sets of primers (Table S3), based on the MiFish fish-universal primer framework reported by Miya et al. with slight modifications [13]. Full reagent conditions, thermal cycling conditions, QC procedures, and bioinformatics parameters are provided in Supplementary Methods. Briefly, we performed the first PCR with KAPA HiFi HotStart ReadyMix (KAPA Biosystems), and MiFish primers. Following the purification of the initial PCR products using the SPRI beads (Beckman Coulter), each DNA sample underwent four replicate PCRs. We normalized DNA concentrations to 0.1 ng/µL before library preparation; negative controls were diluted using the mean dilution factor applied to samples. We set up the index PCR in 12 µL, using KAPA HiFi HotStart ReadyMix and 1 µL of first-round PCR product. The pooled library was then size-selected to enrich the expected fragment length (∼370 bp), checked for quality, normalized, and sequenced with paired-end reads. We normalized libraries to 1 nM and sequenced them on an Illumina iSeq 100 platform (2 × 150 bp paired-end). We processed iSeq reads in USEARCH v10 following the established MiFish pipeline, including read merging, primer trimming, quality filtering, dereplication, and UNOISE3 denoising to generate ASVs [14]. We assigned taxonomy against MitoFish (v3.90) and removed non-target assignments prior to downstream analyses. Field blanks and no-template controls were used to assess potential contamination. Taxon-specific reads detected in negative controls were subtracted from the corresponding environmental samples before downstream analyses.

#### Data analysis

All data analyses were run in R ver. 4.3.3 [15], employing vegan (v2.5–6) [16] and lme4 (v1.1–21) [17] as the main analysis packages. We tested for differences in eDNA yield and detected species richness among filtration methods using one-way ANOVA and we applied Tukey's test. The strength of correlations between the eDNA yields and the sample volume was determined based on Pearson's correlation coefficients (*r*). We rarefied read counts to the minimum depth across samples using vegan and performed downstream analyses on the rarefied dataset (Fig. S1). For metabarcoding outputs, we evaluated in three ways: species richness, within-method β diversity, and comparison of fish species between the listed and detected species. We examined differences in metabarcoding-based species richness among concentration methods after converting read data to presence–absence values. To assess how concentration methods influenced β diversity among replicate filtrations, we used pseudo β diversity, defined here as β diversity calculated between independent replicates derived from the same source sample. We computed pseudo β diversity using the same settings as described previously [9]. Several plots were produced using ggplot2 (v3.1.1). To assess sequencing-depth-dependent taxon accumulation and the extent to which richness approached saturation, we additionally generated abundance-based, sample-size-based rarefaction and extrapolation curves from unrarefied post-quality-control read counts using iNEXT v3.0.2 in R v4.3.3 [18]. For each experiment, read counts from the four biological replicates were pooled by taxon within each concentration method-volume condition. The iNEXT analysis was used descriptively at the pooled condition level and was not used for statistical comparisons of replicate-level species richness among concentration methods. Curves were calculated for Hill number *q* = 0 with 40 knots and extrapolated to twice the observed read count; 95% confidence intervals were estimated from 50 bootstrap replications (Fig. S2). Read counts were treated as sequencing observations for evaluating taxon accumulation and sampling completeness, not as estimates of organismal abundance.

## Experiment 2: comparison of QCV, GF, and Sterivex using natural seawater

### Sample collection of natural seawater

Surface seawater was sampled at the Kobe Port (34° 38′27.9″ N, 135° 13′35.8″ E) on November 6, 2024. To evaluate the scalability and efficiency of each method, we filtered 0.5 L, 1.0 L, 2.0 L, and 5.0 L, although the 5.0 L volume was processed only with QCV and GF because suspended solids and fine particles in the seawater caused clogging of the Sterivex filter. We prepared a set of four independent replicates and a field blank. Benzalkonium chloride was added to each sample (final 0.001% w/v), and the samples were immediately processed using QCV, GF, or Sterivex.

### Filtration and extraction

Procedures were based on Experiment 1, with slight changes as noted below. After the nucleic acid purification, the extracts were further purified using DNeasy PowerClean Pro Cleanup Kit (Qiagen). We eluted DNA in 100 µL AE buffer and measured DNA yields.

### qPCR reaction

We quantified *Acanthopagrus schlegelii* eDNA in seawater by species-specific qPCR to assess how concentration methods affected eDNA yields. This species was chosen because earlier studies verified their abundant presence in this environment. Based on early studies [19], we selected established primer and probe designs to support specific and dependable amplification (Table S1A). The qPCR reactions were performed under the same conditions as Experiment 1.

### Metabarcoding analysis for experiment 2

We performed fish eDNA metabarcoding to compare concentration methods in terms of detected species richness and community composition. For the metabarcoding analysis, the three methods were evaluated using only the 0.5 L and 2.0 L sample volumes, as filtering 5.0 L with Sterivex was difficult and did not yield nucleic acids. Bioengineering Lab Co., Ltd. (Kanagawa, Japan) prepared amplicon libraries using a two-step tailed PCR workflow with four MiFish-derived primer sets (Table S3). Full reagent conditions, PCR conditions, QC procedures, and bioinformatics parameters are provided in Supplementary Methods. The first-round PCR amplified fish eDNA from each extract, and amplicons were purified using magnetic beads prior to a second-round PCR. Libraries were quality-checked and pooled for paired-end sequencing on an Illumina MiSeq platform (2 × 300 bp, the Miseq reagent kit v3). Sequence reads were processed using an established MiFish pipeline, including demultiplexing, primer trimming, quality filtering, denoising to infer amplicon sequence variants (ASVs), and taxonomic assignment against the MitoFish mitochondrial database.

### Data analysis

We used the same procedures as in Experiment 1, excluding the comparative analysis against the known species list.

## Method validation

### Experiment 1: An evaluation of QCV using aquarium tank water

To evaluate the linearity between sample volume and eDNA yield, and to validate detection capabilities using a known species composition, we collected water samples from four aquarium tanks at Hakkeijima Sea Paradise. According to an unpublished report from the aquarium, the four tanks harbored a diverse range of 155 fish species at the time of sampling. We compared three concentration methods: GF, Sterivex, and QCV. Sample volumes of 0.5 L, 1.0 L, and 2.0 L were processed to assess the relationship between filtration volume and eDNA recovery. The overall copy numbers are shown in Table S4A. None of the qPCR field blanks or no-template controls produced an exponential amplification curve crossing the predefined detection threshold across all runs.

**Fig. 2:**
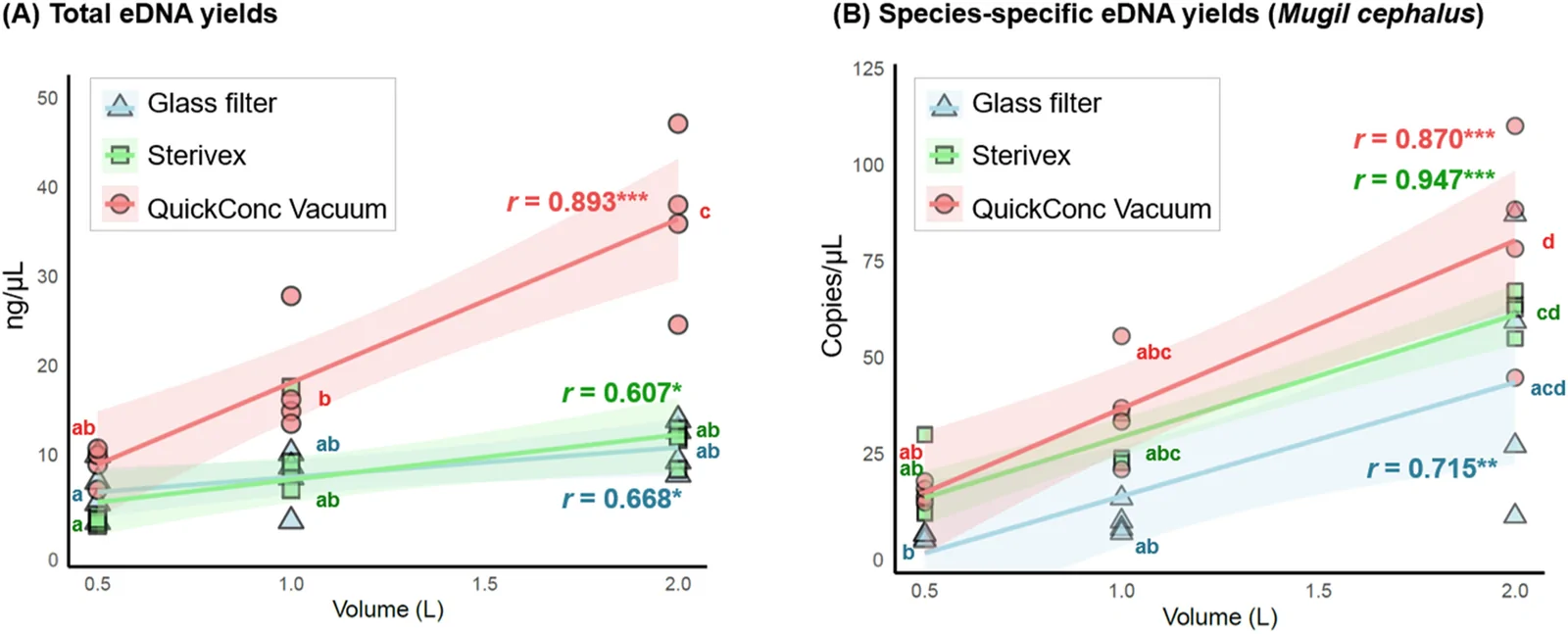
Total and species-specific eDNA yields using aquarium tank water. The experiment was conducted using 0.5 L, 1.0 L, and 2.0 L of aquarium tank water concentrated using QuickConc Vacuum (QCV), the glass filter (GF) and Sterivex methods. Total eDNA yields (A), and *Mugil cephalus* eDNA (B) are summarized for glass fiber filter (GF), Sterivex, and QuickConc Vacuum (QCV) across filtration volumes. The point represents a sample illustrated by method as follows: GF in blue, Sterivex in green, and QCV in red. Shaded areas indicate the 95% confidence intervals. Pearson's correlation coefficients (*r*) were calculated using sample volume and eDNA yields. Statistically significant Pearson's correlation coefficients are listed in the graph (*p < 0.05, ^⁎⁎^*p* < 0.01, and ^⁎⁎⁎^*p* < 0.001). Different letters indicate statistically significant differences among factor levels (Tukey’s HSD, *p* < 0.05).(For interpretation of the references to color in this figure legend, the reader is referred to the web version of this article.).

The total eDNA and the species-specific eDNA yields increased in significantly positive correlation with the sample volume for all three methods (Fig. 2A, B) (Pearson’s correlation of total eDNA and species-specific eDNA yields; QCV *r* = 0.893 and 0.870, GF *r* = 0.668 and 0.715, and Sterivex *r* = 0.607 and 0.947). QCV consistently yielded higher total eDNA yields compared to GF and Sterivex across all volumes (0.5–2.0 L). Specifically, QCV showed a significantly higher total eDNA in the 2 L sample. We further quantified species-specific eDNA targeting *Mugil cephalus*, a species present in the aquarium tank. The copy numbers of *M. cephalus* DNA increased linearly with filtration volume in each method; however, there were no significant differences in the quantities of species-specific eDNA among the methods at each sample volume.

**Fig. 3:**
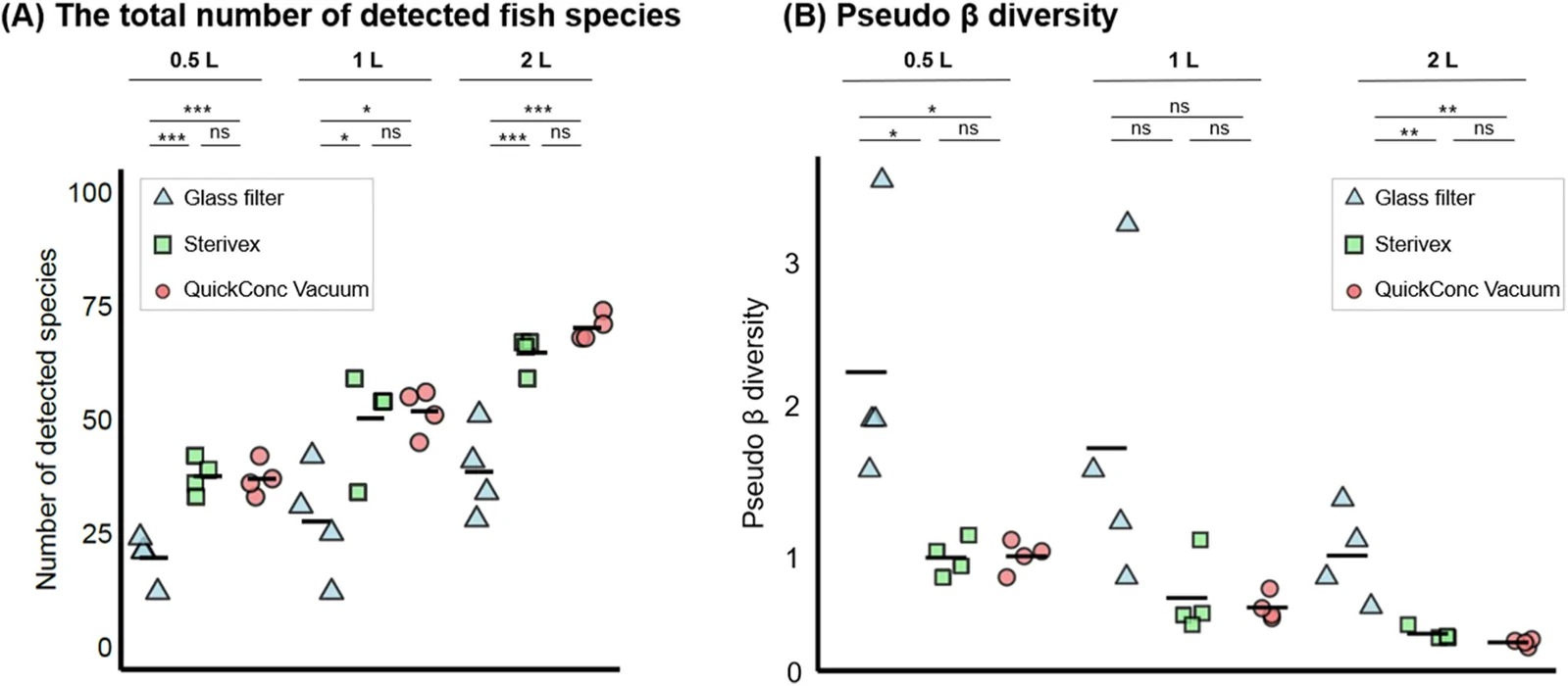
Species richness and within-method variability using aquarium tank water. Fish species richness (A) and pseudo β diversity (B) are summarized for glass fiber filter (GF), Sterivex, and QuickConc Vacuum (QCV) across filtration volumes. The point represents individual replicates illustrated by method as follows: GF in blue, Sterivex in green, and QCV in red; the midlines indicate the averages. Differences among groups were examined using Tukey’s test (^ns^*p* ≧ 0.05, *p < 0.05, ^⁎⁎^*p* < 0.01, ^⁎⁎⁎^*p* < 0.001).(For interpretation of the references to color in this figure legend, the reader is referred to the web version of this article.).

**Fig. 4:**
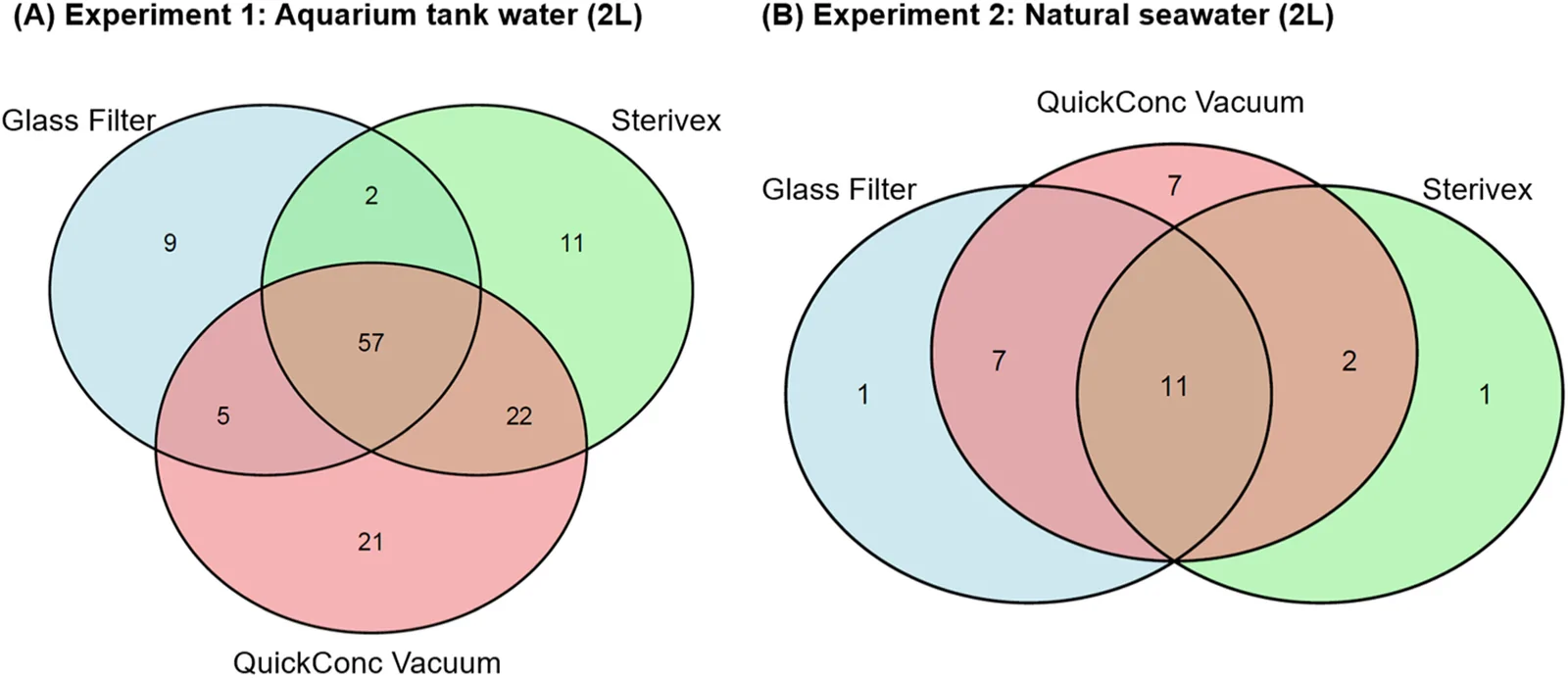
Venn diagrams of fish species detected by each method. These show the overlap of fish species detected by three methods in aquarium tank water (A) and natural seawater (B). The number of detected species was pooled from four replicates for each condition. Glass fiber filter, Sterivex, and QuickConc Vacuum are shown in blue, green, and red, respectively, using 2 L water samples for each method.(For interpretation of the references to color in this figure legend, the reader is referred to the web version of this article.).

We assessed fish species richness by MiFish-based metabarcoding (Table S5). No sequence reads assigned to fish taxa were detected in any field blank or no-template control. The richness of identified fish increased with sample volume for all methods (Fig. 3A), and QCV and Sterivex detected more species than GF across volumes. To examine whether filtration approach alters within-method community variability, we calculated pseudo β diversity for each concentration method (Fig. 3B). While all methods showed a decreasing trend in pseudo β diversity with increasing sample volume, QCV and Sterivex exhibited significantly lower values than GF at 0.5 L and 2 L. In the aquarium samples at the 2 L volume, 57 species were commonly detected by all three methods (Fig. 4A). QCV yielded the highest total number of species (105), followed by Sterivex (92) and GF (73). Additionally, QCV identified 21 unique species not found by the other methods, whereas Sterivex and GF detected 11 and 9 unique species, respectively. Sample-size-based rarefaction and extrapolation curves approached plateaus for all method-volume conditions, indicating limited additional taxon recovery when sequencing depth was extrapolated to twice the observed read count (Fig. S2A).

To evaluate the validity of the detected species, we compared the metabarcoding results with the known species composition of the aquarium. Out of the 155 listed fish species in the aquarium tank, the number of detected species increased with sample volume for all three methods (Table 1 and S6). At the maximum volume of 2 L, 84 of 155 species (54%) were detected by QCV, 81 species (52%) by Sterivex, and 64 s species (41%) by GF. In addition to the listed species, several species not recorded in the list were also detected. To identify their origins, aquarium staff classified these species into three categories: "feeds and natural seawater," "other tanks," and "unknown sources." Based on this detailed classification performed by the aquarium staff, it was determined that a significant proportion of these non-listed species were derived from feeds or the intake natural seawater. Notably, detections attributed to species from other tanks were minimal, demonstrating that cross-contamination was effectively prevented throughout the sampling and filtration processes.

**Table 1:** Overview of sequencing reads and species richness for aquarium water samples.

	Glass filter			Sterivex			QuickConc Vacuum		
	0.5 L	1 L	2 L	0.5 L	1 L	2 L	0.5 L	1 L	2 L
Total read count*(after assignment to species)	92,796	100,611	97,505	88,588	86,340	133,631	172,925	159,704	188,740
Tank fish	92,071	90,692	96,141	87,220	84,292	131,824	165,727	150,358	154,969
Non-tank fish	725	9919	1364	1368	2048	1807	7198	9346	33,771
Total number of tank species	155								
number of tank fish (ratio)	37(24%)	49(32%)	64(41%)	56(36%)	70(45%)	81(52%)	61(39%)	75(48%)	84(54%)
number of fish out of the list	4	6	9	4	14	11	7	8	21
feeds and natural seawater	4	6	8	3	10	10	5	8	15
other tanks	0	0	1	1	3	0	0	0	2
unknown sources	0	0	0	0	1	1	2	0	4

*The results are compared among glass filter, Sterivex, and QuickConc Vacuum with three different filtration volumes. Read counts were pooled from four replicates for each condition. The detection rates relative to the total number of known tank species (155) are shown in parentheses.

### Experiment 2: assessment of QCV, GF, and Sterivex using seawater

To demonstrate the applicability of QCV in a natural environment, we gathered surface seawater samples from Kobe Port. Sample volumes of 0.5 L, 1.0 L, 2.0 L, and 5.0 L were processed to assess the relationship between filtration volume and eDNA recovery, except for the 5.0 L sample for Sterivex, due to clogging. The overall copy numbers are shown in Table S4B. None of the qPCR field blanks or no-template controls produced an exponential amplification curve crossing the predefined detection threshold across all runs.

**Fig. 5:**
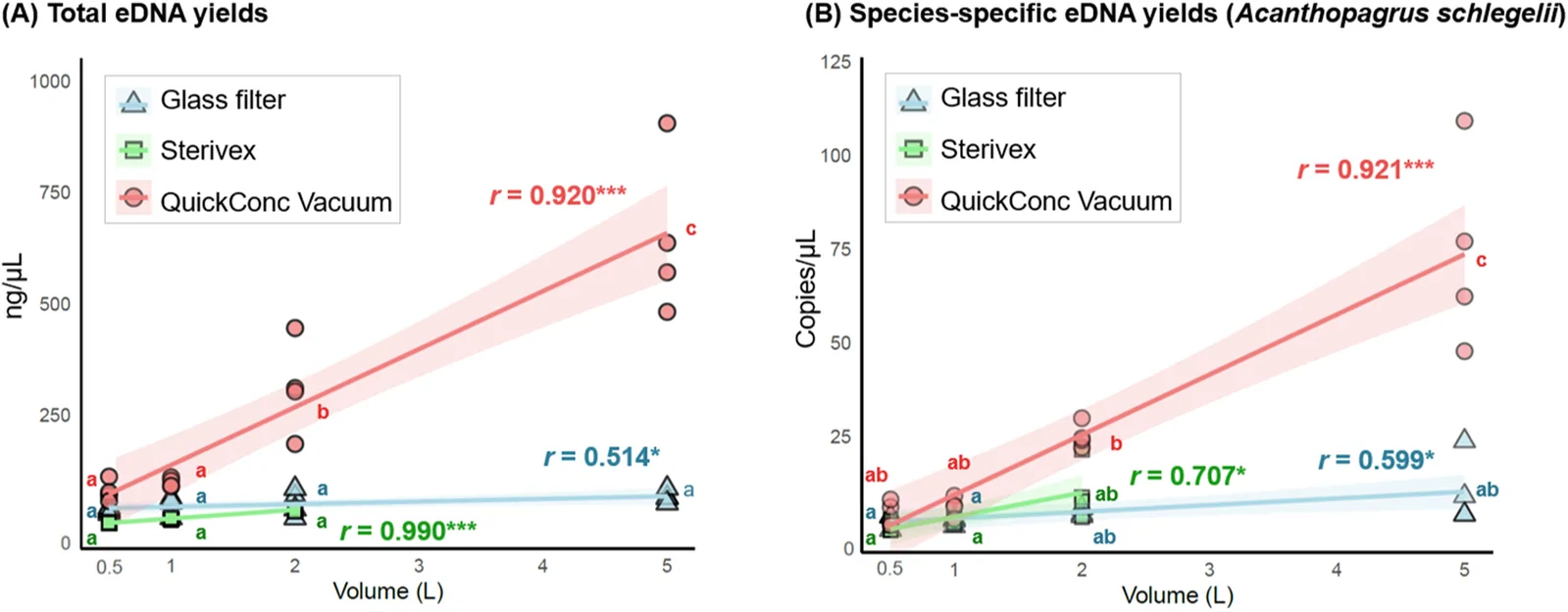
Total and species-specific eDNA yields using natural seawater. Using natural seawater (0.5, 1.0, 2.0, and 5.0 L), we compared eDNA yields among QuickConc Vacuum (QCV), the glass fiber filter (GF) and Sterivex methods. Total eDNA yields and *Acanthopagrus schlegelii* eDNA are shown in panels A and B, respectively. The point represents individual replicates illustrated by method as follows: GF in blue, Sterivex in green, and QCV in red. Shaded areas indicate the 95% confidence intervals. Pearson's correlation coefficients (*r*) were calculated using sample volume and eDNA yields. Statistically significant Pearson's correlation coefficients are listed in the graph (*p < 0.05 and ^⁎⁎⁎^*p* < 0.001). Different letters indicate statistically significant differences among factor levels (Tukey’s HSD, *p* < 0.05).(For interpretation of the references to color in this figure legend, the reader is referred to the web version of this article.).

For each concentration technique, a strong linear relationship was established between the filtered volume and the resulting eDNA yields (Fig. 5A, B) (Pearson’s correlation; QCV *r* = 0.920 and 0.921, GF *r* = 0.514 and 0.599, and Sterivex *r* = 0.990 and 0.77). QCV yielded higher total eDNA than GF and Sterivex, particularly at 2.0 L and 5.0 L, and it also produced higher *Acanthopagrus schlegelii* copy numbers at 5.0 L. Notably, QCV maintained high linearity and filtration capability even at larger volumes up to 5 L, whereas conventional methods often faced clogging or lower recovery rates per unit volume. However, for natural seawater, additional purification was implemented. While larger volumes increase eDNA yields, they may also concentrate PCR inhibitors.

**Fig. 6:**
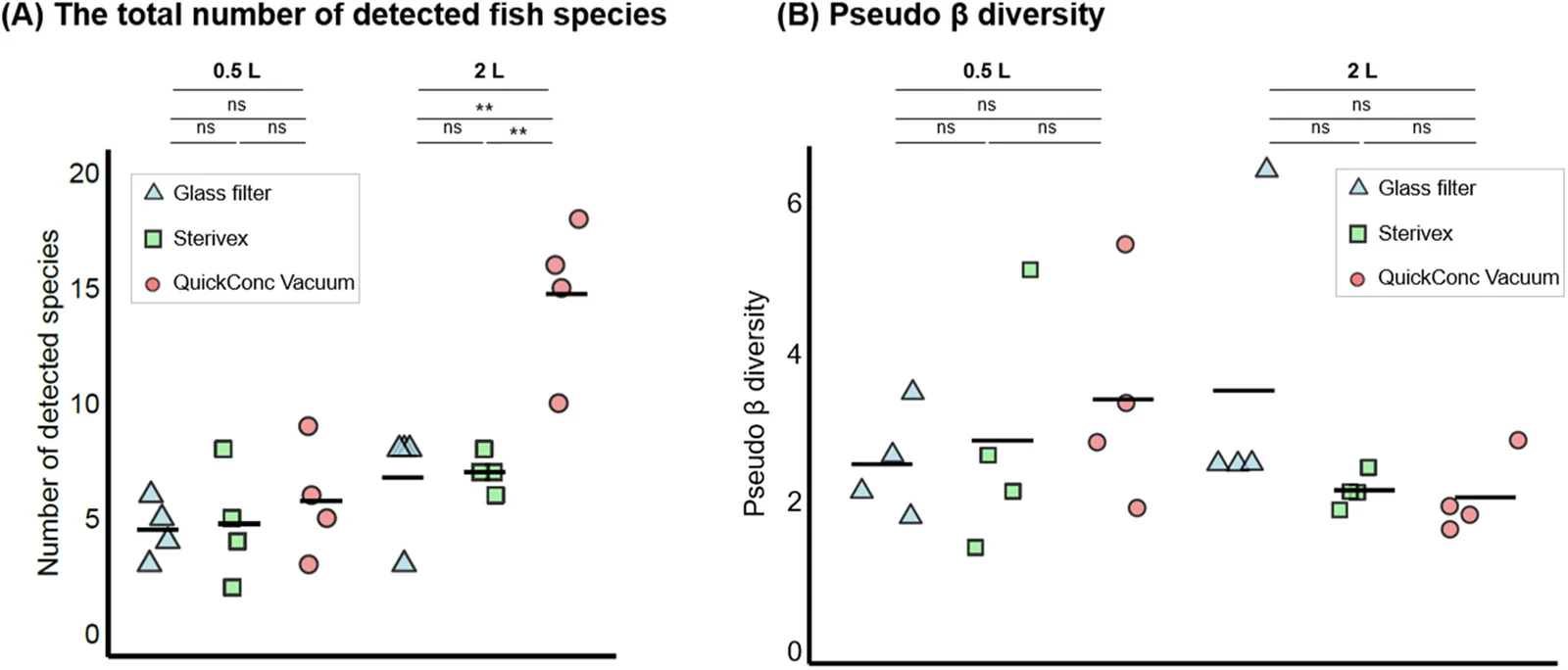
Fish species richness and within-method variability using natural seawater. Panels A and B summarize fish species richness and pseudo β diversity, respectively, for glass fiber filter (GF), Sterivex, and QuickConc Vacuum (QCV). The point represents a sample illustrated by method as follows: GF in blue, Sterivex in green, and QCV in red; the midlines indicate the averages. Between-group differences were evaluated using Tukey's honest significant difference test (^ns^*p* ≧ 0.05, *p < 0.05, ^⁎⁎⁎^*p* < 0.001). ns: not significant.(For interpretation of the references to color in this figure legend, the reader is referred to the web version of this article.).

In the metabarcoding analyses of the seawater samples, no sequence reads assigned to fish taxa were detected in any field blank or no-template control. There were no significant differences in the number of detected species among the methods at the 0.5 L volume, whereas QCV detected more species than GF and Sterivex at 2.0 L (Fig. 6A). Within-method community variability (pseudo β diversity) within each method was comparable among methods at both 0.5 L or 2 L sample volumes (Fig. 6B). The ability of QCV to process 2 L of seawater combined with its high eDNA yields resulted in the detection of a broader range of biodiversity, likely including rare or low-density species that were missed by conventional filtration. In the natural seawater samples at the 2 L volume, 11 species were shared across all methods (Fig. 4B). QCV detected seven unique species, while the GF and Sterivex methods each detected only one unique species. These results indicate that QCV consistently detected a higher number of unique species in both experiments. The corresponding curves also approached plateaus for all method-volume conditions, indicating limited additional taxon recovery when sequencing depth was extrapolated to twice the observed read count (Fig. S2B).

### Limitations

QCV performance and the maximum filterable volume depend on water conditions; highly turbid water can cause clogging and reduce throughput. Larger volumes can also co-concentrate PCR inhibitors, and additional purification may be required for some natural seawater samples. Method selection should consider both analytical performance and practical constraints. QCV is suitable when large-volume processing and high detection sensitivity are required, whereas GF provides a relatively low-cost option for processing large water volumes [20]. Previous comparisons indicate that vacuum-filtration approaches can be more cost-effective than Sterivex filtration, although actual per-sample costs vary depending on suppliers, regions, and protocol-specific consumables. Sterivex is well suited for on-site filtration, and its closed-cartridge format can reduce sample handling and the risk of contamination; however, clogging prevented processing of 5.0 L seawater samples in this study. Therefore, the optimal method depends on sample volume, water characteristics, required sensitivity, available equipment, and cost.

## Ethics statements

No ethical approvals or considerations were necessary for this study.

## CRediT author statement

Tomohiro Kuroita: Conceptualization, validation, investigation, data curation, writing – original draft. Qianqian Wu: Investigation, data curation, writing – review & editing. Ryo Iwamoto: Conceptualization, writing – review & editing, project administration. Toshifumi Minamoto: Conceptualization, writing – review & editing, project administration, funding acquisition.

## Declaration of interests

The authors declare the following financial interests/personal relationships which may be considered as potential competing interests: The authors disclose the following interests that could be perceived as competing: T.K. and R.I. are employees of Shionogi & Co., Ltd. and AdvanSentinel Inc., and are inventors on patents related to QuickConc. T.M. is an inventor on a patent covering the use of benzalkonium chloride for eDNA preservation.

## Data Availability

Data will be made available on request.

## Appendix Supplementary materials

Image, application 1
