# Supplementary material for: QuickConc Vacuum: A high-sensitive cationic-assisted method for large-volume eDNA concentration and sensitive biodiversity monitoring
Source: MethodsX. 2026 Aug 27;17:104126. doi: 10.1016/j.mex.2026.104126 (PMC13578458; doi:10.1016/j.mex.2026.104126)
Supplement: Supplementary file 1 [file mmc1.docx]

**Supplementary information**

**Supplementary Methods**

**Amplicon library preparation, MiSeq sequencing, and data processing for Experiment 1**

The first-round PCR reaction was performed in a 12 μL reaction mixture containing 6.0 μL of 2× KAPA HiFi HotStart ReadyMix (KAPA Biosystems, Wilmington, MA, USA), 3.6 pmol of each primer, 2 μL of the eDNA template, and ultrapure water to adjust the final volume. The thermal cycle profile was 95 °C for 3 min, followed by 40 cycles of 20 s at 98 °C, 15 s at 65 °C, and 15 s at 72 °C, with a final extension of 5 min at 72 °C. Each DNA sample was processed in four replicate PCRs after purification of the first PCR products using the SPRI Select Reagent Kit (Beckman Coulter, Brea, CA, USA) according to the manufacturer’s protocol. All samples were diluted to a concentration of 0.1 ng/μL, and negative controls were diluted based on the average dilution ratio applied to the samples [11]. The second-round PCR had a 12 μL total reaction volume, including 6.0 μL 2× KAPA HiFi HotStart ReadyMix (KAPA Biosystems), 3.6 pmol of each primer, 1 μL of the first PCR product, and ultrapure water. The second PCR’s thermal cycles profile consisted of 95 °C for 3 min, 12 cycles at 98 °C for 20 s, and 72 °C for 20 s, with a final extension at 72 °C for 5 min. The second PCR products were pooled into a single tube to encompass all samples. Target-sized amplicons were isolated using the E-Gel Precast Agarose Electrophoresis System (Thermo Fisher Scientific) with an E-Gel SizeSelect™ 2% agarose gel. To confirm the selective recovery of DNA fragments of the intended length (~370 bp), the pooled library was subsequently assessed on an Agilent 2100 Bioanalyzer (Agilent Technologies). The DNA library was normalized to 1 nM and sequenced on an Illumina iSeq 100 System with 2 × 150 bp paired-end chemistry. First, paired-end reads (R1 and R2) were assembled using the command “-fastq_mergepairs”. Second, the primer sequences were removed from both edges of the assembled reads using the commands “-fastx_truncate”, “-stripleft”, and “-stripright”. Third, low-quality reads with an expected error rate of > 1% and reads shorter than 140 bases were removed by quality filtering using the command “-fastq_filter”. Fourth, the preprocessed reads were dereplicated using the “fastx_uniques” command. Finally, the dereplicated reads were denoised using the “unoise3” command to generate amplicon sequence variants (ASVs) by removing all putatively chimeric and erroneous sequences. For MiFish analyses, the obtained ASV table was analyzed with USEARCH v 10.0.240 (Edgar, 2010), according to the method demonstrated by Wu, Sakata et al. [14]. The mitochondrial genome database MitoFish (ver. 3.90) was used to assign taxonomic names to ASVs.

**Amplicon library preparation, MiSeq sequencing, and data processing for Experiment 2**

The first-round PCR reaction was conducted in a 10 μL mixture containing 0.375 U ExTaq HS (TaKaRa), 1× ExTaq Buffer, 0.2 mM of each dNTPs, 0.5 μM of each primer, 1 μL of the eDNA template, and ultrapure water. The thermal cycle profile was 94 °C for 2 min, followed by 35 cycles of 20 s at 94 °C, 15 s at 65 °C, and 20 s at 72 °C, with a final extension of 5 min at 72 °C. After purification of the first PCR products using VAHTS DNA Clean Beads (Vazyme), the second-round PCR had a 10 μL total reaction volume, including 0.2 U KOD FX Neo (TOYOBO), 1 × PCR Buffer, 0.4 mM of each dNTPs, 0.25 μM each primer, 2 ng of the first PCR product, and ultrapure water. The second PCR’s thermal cycles profile consisted of 94 °C for 2 min, 12 cycles at 98 °C for 10 s, 60 °C for 30 s, and 68 °C for 30 s, with a final extension at 68 °C for 2 min. PCR products were purified using VAHTS DNA Clean Beads (Vazyme), and then the paired-end sequence libraries were completed. The quality of the constructed library was verified using a fragment analyzer and the dsDNA 915 Reagent Kit (Agilent Technologies), after which sequencing was conducted on the MiSeq system using the MiSeq Reagent Kit v3 (Illumina) under conditions of 2 × 300 bp. Only read sequences, which were fully matched with primer sequences at the beginning, were extracted using the fastq barcode splitter in the FASTX Toolkit (ver. 0.0.14). FASTX-Toolkit's fastx_trimmer was used to remove the primer sequences and the last 120 bases at the 3’ end from the extracted reads. Subsequently, sequences with a quality score below 20 were filtered out using Sickle (ver. 1.33), and any sequences shorter than 40 bases, along with their paired reads, were discarded. Following this, the DADA2 plugin in Qiime2 (ver. 2024.2) was used to remove chimeric and noisy sequences, after which representative sequences and an ASV table were generated. For MiFish analyses, the obtained ASV table was analyzed with USEARCH v 10.0.240 (Edgar, 2010), according to the method demonstrated by Wu, Sakata et al. [14]. The mitochondrial genome database MitoFish (ver. 3.90) was used to assign taxonomic names to ASVs.

**Sample-size-based rarefaction and extrapolation analysis**

To evaluate sequencing-depth-dependent taxon accumulation and the extent to which richness approached saturation, unrarefied post-quality-control metabarcoding read counts were analyzed using iNEXT v3.0.2 in R v4.3.3 [18]. For each experiment, read counts from four biological replicates were pooled by taxon within each concentration method-volume condition. Surrounding whitespace in taxon labels was removed, and exact duplicate taxon labels were combined before analysis. Abundance-based sample-size curves were calculated for Hill number q = 0 using 40 knots and extrapolated to twice each condition's observed read count. The 95% confidence intervals were estimated using 50 bootstrap replications with a fixed random seed. Read counts were treated as sequencing observations for evaluating taxon accumulation and sampling completeness, not as estimates of organismal abundance. Asymptotic diversity estimates were not calculated or interpreted; only the rarefaction and extrapolation curve estimates were used in this analysis.

Table S1.

(A) Nucleotide sequences of primers and probes

FAM: 6-carboxyfluorescein; TAMRA: Tetramethylrhodamine

| Assay | Primer/Probe | Name | Sequence (5’-3’) | Reference |
| --- | --- | --- | --- | --- |
| *Mugil cephalus cephalus* | Forward | Mce-CytB-F | AACAACCCCCTCGGATTACC |  |
|  | Reverse | Mce-CytB-R | GTGCGAGGGAGATGAGTGTAAAG | (Yamanaka and Minamoto., 2016) |
|  | Probe | Mce-CytB-P | FAM-CAACTCAGACAAAATCCCCTTTCACCCC-TAMRA |  |
| *Acanthopagrus schlegelii* | Forward | Asc_Cytb_F | CTGTCTGCCGTCCCCTACA |  |
|  | Reverse | Asc_Cytb_R | TATGGCGGCTACGATAAAAGGA | (Takahashi et al., 2020) |
|  | Probe | Asc_Cytb_Pr | FAM-TCAGTTGACAACGCAACCCTAACCCG-TAMRA |  |

(B) Components of the reaction mixture for qPCR

| Component | Volume (μL) |
| --- | --- |
| 2× Environmental Master Mix 2.0 | 10 |
| AmpErase Uracil N-Glycosylase | 0.1 |
| 10μM Primer Mix | 1.8 |
| 10μM Probe | 0.25 |
| RNase-free water | 5.85 |
| eDNA extract | 2 |
| Total | 20 |

Table S2. R^2^ values, slopes, and Y intercepts of the calibration curves, and the PCR efficiencies for each target species.

| **Experiment** | **Target species** | **R^2^ value** | **Slope** | **Y-Intercepts** | **PCR efficiency%** |
| --- | --- | --- | --- | --- | --- |
| 1 | *Mugil cephalus cephalus* | 0.99 | -3.24 | 38.4 | 103 % |
| 2 | *Acanthopagrus schlegelii* | 0.99 | -3.35 | 40.0 | 99 % |

Table S3. Nucleotide sequences of metabarcoding primers

| PCR | Primer | name | Sequence (5-3’) | Reference |
| --- | --- | --- | --- | --- |
| 1st^1^ | Forward | MiFish-E-F-v2 | ACACTCTTTCCCTACACGACGCTCTTCCGATCTNNNNNNRGTTGGTAAATCTCGTGCCAGC | (The eDNA Society, 2019) |
|  | Reverse | MiFish-E-R-v2 | GTGACTGGAGTTCAGACGTGTGCTCTTCCGATCTNNNNNNGCATAGTGGGGTATCTAATCCTAGTTTG |  |
|  | Forward | MiFish-U-F | ACACTCTTTCCCTACACGACGCTCTTCCGATCTNNNNNNGTCGGTAAAACTCGTGCCAGC | (Miya et al., 2015) |
|  | Reverse | MiFish-U-R | GTGACTGGAGTTCAGACGTGTGCTCTTCCGATCTNNNNNNCATAGTGGGGTATCTAATCCCAGTTTG |  |
|  | Forward | MiFish-U2-F | ACACTCTTTCCCTACACGACGCTCTTCCGATCTNNNNNNGCCGGTAAAACTCGTGCCAGC | (The eDNA Society, 2019) |
|  | Reverse | MiFish-U2-R | GTGACTGGAGTTCAGACGTGTGCTCTTCCGATCTNNNNNNCATAGGAGGGTGTCTAATCCCCGTTTG |  |
|  | Forward | MiFish-Ayu&Wakasagi | ACACTCTTTCCCTACACGACGCTCTTCCGATCTGCCGGTTAATCTCGTGCCAGC | (Bioengineering Lab. Co. , Ltd., 2020) |
|  | Reverse | MiFish-Ayu&Wakasagi | GTGACTGGAGTTCAGACGTGTGCTCTTCCGATCTCATAGTGGGGTATCTAATCCCAGTTTG |  |
| 2nd | Forward | 2nd F | AATGATACGGCGACCACCGAGATCTACAC-Index-ACACTCTTTCCCTACACGACGC | (Miya et al., 2015) |
|  | Reverse | 2nd R | CAAGCAGAAGACGGCATACGAGAT-Index-GTGACTGGAGTTCAGACGTGTG |  |

^1^For the 1st PCR, MiFish-U-F/R was used in Experiment 1. In Experiment 2, a primer mix was prepared in the following ratio: MiFish-E-F/R :MiFish-U-F/R: MiFish-U2-F/R: MiFish-Ayu&Wakasagi = 2: 4: 2: 1.

Table S4. Quantitative real-time PCR (qPCR) for each target species with different experiments.

(A) Experiment 1

| **Method** | **Sample**  **volume (L)** | **ID** | **Target species** |
| --- | --- | --- | --- |
|  |  |  | ***Mugil cephalus cephalus*** |
|  |  |  | **eDNA concentration**  **(copies/ μ L)** |
| Glass filter | 0.5 | 1 | 4 |
|  | 0.5 | 2 | 2.8 |
|  | 0.5 | 3 | 2.3 |
|  | 0.5 | 4 | 3.9 |
|  | 1 | 1 | 7.4 |
|  | 1 | 2 | 13.4 |
|  | 1 | 3 | 5.5 |
|  | 1 | 4 | 4.4 |
|  | 2 | 1 | 87.1 |
|  | 2 | 2 | 27 |
|  | 2 | 3 | 8.7 |
|  | 2 | 4 | 59.3 |
| Sterivex | 0.5 | 1 | 29.9 |
|  | 0.5 | 2 | 13.5 |
|  | 0.5 | 3 | 9.8 |
|  | 0.5 | 4 | 9.4 |
|  | 1 | 1 | 35.4 |
|  | 1 | 2 | 22.8 |
|  | 1 | 3 | 23.8 |
|  | 1 | 4 | 22.5 |
|  | 2 | 1 | 63.7 |
|  | 2 | 2 | 54.9 |
|  | 2 | 3 | 67.2 |
|  | 2 | 4 | 62.5 |
| QuickConc  Vacuum | 0.5 | 1 | 13.7 |
|  | 0.5 | 2 | 12.3 |
|  | 0.5 | 3 | 15.8 |
|  | 0.5 | 4 | 17.8 |
|  | 1 | 1 | 55.5 |
|  | 1 | 2 | 36.8 |
|  | 1 | 3 | 33.3 |
|  | 1 | 4 | 20.9 |
|  | 2 | 1 | 44.7 |
|  | 2 | 2 | 78.2 |
|  | 2 | 3 | 88.4 |
|  | 2 | 4 | 110.1 |

(B) Experiment 2

| **Method** | **Sample**  **volume (L)** | **ID** |  |
| --- | --- | --- | --- |
|  |  |  | ***Acanthopagrus schlegelii*** |
|  |  |  | **eDNA concentration**  **(copies/ μ L)** |
| Glass filter | 0.5 | 1 | 0 |
|  | 0.5 | 2 | 3.6 |
|  | 0.5 | 3 | 3.6 |
|  | 0.5 | 4 | 3.6 |
|  | 1 | 1 | 2.8 |
|  | 1 | 2 | 3.1 |
|  | 1 | 3 | 1 |
|  | 1 | 4 | 2.5 |
|  | 2 | 1 | 4.3 |
|  | 2 | 2 | 5.9 |
|  | 2 | 3 | 3.7 |
|  | 2 | 4 | 5.8 |
|  | 5 | 1 | 4.1 |
|  | 5 | 2 | 9 |
|  | 5 | 3 | 23.7 |
|  | 5 | 4 | 4.1 |
| Sterivex | 0.5 | 1 | 0 |
|  | 0.5 | 2 | 2.7 |
|  | 0.5 | 3 | 0 |
|  | 0.5 | 4 | 1.1 |
|  | 1 | 1 | 2.7 |
|  | 1 | 2 | 3.3 |
|  | 1 | 3 | 1.5 |
|  | 1 | 4 | 2 |
|  | 2 | 1 | 21.5 |
|  | 2 | 2 | 8.6 |
|  | 2 | 3 | 3.5 |
|  | 2 | 4 | 7.6 |
| QuickConc  Vacuum | 0.5 | 1 | 2 |
|  | 0.5 | 2 | 6.1 |
|  | 0.5 | 3 | 8.1 |
|  | 0.5 | 4 | 1.2 |
|  | 1 | 1 | 9.1 |
|  | 1 | 2 | 6.1 |
|  | 1 | 3 | 6.5 |
|  | 1 | 4 | 2.9 |
|  | 2 | 1 | 22 |
|  | 2 | 2 | 29.8 |
|  | 2 | 3 | 23.9 |
|  | 2 | 4 | 24.5 |
|  | 5 | 1 | 62.3 |
|  | 5 | 2 | 77 |
|  | 5 | 3 | 109.2 |
|  | 5 | 4 | 47.7 |

Table S5. Detailed list of fish species detected from different concentration methods by eDNA metabarcoding

**Experiment 1**

(A) Glass Filter

| Species name | 0.5 L | | | | 1 L | | | | 2 L | | | |
| --- | --- | --- | --- | --- | --- | --- | --- | --- | --- | --- | --- | --- |
|  | 1 | 2 | 3 | 4 | 1 | 2 | 3 | 4 | 1 | 2 | 3 | 4 |
| Total read count | 30,873 | 32,099 | 19,916 | 27,469 | 28,425 | 26,182 | 33,982 | 27,505 | 29,262 | 28,382 | 35,243 | 27,712 |
| Total read count (after assignment to species) | 27,309 | 26,475 | 17,103 | 21,909 | 24,213 | 21,845 | 30,847 | 23,706 | 24,479 | 23,240 | 27,860 | 21,926 |
| *Acanthurus dussumieri* | 463 |  |  | 1,027 |  | 194 |  |  |  |  |  |  |
| *Acanthopagrus schlegelii/Acanthopagrus sivicolus* |  |  |  |  |  |  |  |  |  | 154 |  | 47 |
| *Acanthurus olivaceus* |  |  |  |  |  |  |  |  |  | 206 | 491 |  |
| *Acanthurus pyroferus* |  |  |  |  |  |  |  |  |  |  |  |  |
| *Acanthurus sohal* |  |  |  |  |  |  |  |  |  |  |  |  |
| *Alectis ciliaris* |  |  |  |  |  | 265 |  |  | 339 |  | 280 | 175 |
| *Alepes kleinii* |  |  |  |  |  |  |  |  |  |  |  |  |
| *Aluterus scriptus* |  |  |  |  | 590 | 357 |  |  |  |  | 1,076 | 110 |
| *Anguilla japonica* |  |  |  |  |  |  |  |  |  |  |  |  |
| *Antennarius commerson* |  |  |  |  |  |  |  |  |  | 199 | 184 | 23 |
| *Apogonichthyoides niger* |  |  |  |  |  |  |  |  |  | 82 |  |  |
| *Arothron hispidus* |  |  |  |  |  |  |  |  |  |  |  |  |
| *Auxis rochei* |  |  |  |  |  |  |  |  |  |  |  |  |
| *Caesio cuning* |  |  |  | 187 |  | 110 | 320 |  |  | 104 | 47 | 28 |
| *Carangoides equula* |  |  |  |  |  |  |  |  |  | 190 |  |  |
| *Centropyge bicolor* |  |  |  |  |  |  |  |  |  |  |  |  |
| *Chaetodon auripes/Chaetodon adiergastos/Chaetodon fasciatus* |  | 541 |  |  | 41 |  | 736 |  |  |  | 426 | 21 |
| *Chaetodon ephippium* |  |  |  |  |  |  |  |  |  |  |  | 66 |
| *Chaetodon kleinii* |  |  |  |  |  |  |  |  |  |  |  |  |
| *Chaetodon lunula* |  |  |  |  |  |  |  |  |  |  |  |  |
| *Chaetodon melannotus* |  |  |  |  |  |  |  |  |  |  |  |  |
| *Cheilodactylus quadricornis/Cheilodactylus zonatus* |  | 459 |  | 435 |  |  |  |  |  |  |  |  |
| *Cheilodactylus zebra/Cheilodactylus quadricornis* | 689 |  |  |  | 539 | 547 |  |  |  | 13 |  | 152 |
| *Chilomycterus reticulatus* | 1,650 | 436 | 2,309 | 3,578 | 3,237 | 728 | 673 |  | 1,202 | 1,494 | 2,624 |  |
| *Cirrhitichthys aureus* | 845 |  |  |  | 186 | 244 | 808 |  |  |  |  | 63 |
| *Clupea pallasii* |  |  |  |  | 222 |  |  |  |  | 31 |  | 40 |
| *Cololabis saira* |  |  |  |  |  |  |  |  |  |  |  |  |
| *Coris aygula* | 166 |  | 3,542 | 132 | 818 | 826 | 1,814 |  | 395 | 810 | 523 | 378 |
| *Cromileptes altivelis* |  |  |  |  |  |  |  |  |  |  |  |  |
| *Ctenochaetus tominiensis* |  |  |  |  | 1,082 |  |  |  |  |  |  |  |
| *Decapterus russelli/Decapterus maruadsi* |  |  |  |  |  | 70 |  |  |  |  |  |  |
| *Dictyosoma burgeri* | 853 |  |  |  |  | 249 |  |  |  |  |  |  |
| *Diodon holocanthus/Diodon liturosus* |  |  |  |  |  | 53 |  |  |  |  | 2,624 |  |
| *Diodon hystrix* |  |  |  |  |  |  |  |  |  |  | 2,624 | 1,357 |
| *Ditrema temminckii temminckii/Ditrema viride* | 426 |  | 476 | 1,338 | 274 | 495 | 1,003 | 691 | 1,150 | 365 | 743 | 286 |
| *Echeneis naucrates* |  |  |  |  |  |  |  |  |  |  |  |  |
| *Eleginus gracilis* |  |  |  |  |  |  |  |  |  |  |  |  |
| *Engraulis ringens* |  |  | 226 |  | 111 |  |  |  |  | 43 |  |  |
| *Enophrys diceraus* |  |  |  |  |  |  |  |  |  |  |  |  |
| *Epinephelus akaara* | 627 | 47 |  | 1,479 | 329 | 828 |  |  | 399 | 367 | 202 | 184 |
| *Epinephelus areolatus* |  | 1,068 | 637 |  | 640 |  | 545 |  |  |  |  | 34 |
| *Epinephelus awoara/Epinephelus coioides* |  | 778 |  |  | 341 | 158 | 431 |  | 274 | 182 | 58 | 345 |
| *Epinephelus coioides/Epinephelus malabaricus* |  |  |  |  |  |  |  |  |  |  |  | 117 |
| *Epinephelus fasciatus* |  |  |  | 877 |  | 88 | 615 |  |  |  |  | 155 |
| *Epinephelus morrhua* |  |  |  | 295 |  |  |  | 1,138 |  |  |  |  |
| *Epinephelus bruneus* |  |  |  |  |  |  |  |  |  |  |  |  |
| *Epinephelus octofasciatus* |  |  |  |  |  |  |  |  |  |  |  |  |
| *Epinephelus septemfasciatus* |  |  |  |  |  |  |  |  |  |  | 76 | 83 |
| *Epinephelus trimaculatus* |  |  |  |  |  |  |  |  |  |  |  |  |
| *Euthynnus alletteratus* |  |  |  |  |  |  |  |  |  |  |  |  |
| *Gadus chalcogrammus/Gadus morhua/Gadus finnmarchicus* |  | 321 |  |  | 191 | 55 |  |  | 269 | 40 | 271 | 258 |
| *Gerres oyena/Gerres equulus* |  |  |  |  |  |  | 97 |  |  | 23 | 34 |  |
| *Girella leonina/Girella melanichthys* |  |  |  |  | 549 |  |  |  |  | 44 |  |  |
| *Girella punctata* |  |  |  |  |  |  |  |  |  |  |  |  |
| *Gymnocanthus herzensteini* |  |  |  |  |  |  |  |  |  |  |  |  |
| *Gymnocranius griseus* |  |  |  |  |  |  |  |  |  |  |  |  |
| *Gymnogobius petschiliensis/Gymnogobius opperiens* |  |  |  |  |  |  |  |  |  |  |  |  |
| *Gymnomuraena zebra* |  |  |  |  |  |  |  |  |  |  |  | 71 |
| *Gymnothorax kidako* | 3,032 | 3,991 | 1,506 | 2,142 | 1,695 | 2,085 | 3,433 |  | 4,412 | 2,457 | 1,906 | 1,869 |
| *Gymnothorax reticularis/Gymnothorax minor* |  |  |  | 511 |  |  |  |  |  |  |  |  |
| *Gymnothorax flavimarginatus* |  |  |  |  |  |  |  |  |  |  |  |  |
| *Halichoeres tenuispinis* |  |  |  |  |  |  |  |  |  |  |  |  |
| *Hapalogenys sennin* |  |  |  |  |  |  |  |  |  | 113 |  |  |
| *Hemitaurichthys polylepis/Hemitaurichthys zoster* |  |  |  | 749 |  | 192 |  |  |  |  |  |  |
| *Hemitripterus villosus* |  |  |  |  |  |  |  |  |  |  |  |  |
| *Heterodontus zebra/Heterodontus japonicus* |  |  |  |  |  |  |  |  |  |  |  |  |
| *Hyporthodus septemfasciatus* |  |  | 863 |  |  | 157 |  |  | 87 |  |  |  |
| *Inimicus japonicus* |  |  |  |  |  |  |  |  | 145 |  |  | 28 |
| *Istigobius campbelli* |  |  |  |  |  |  |  |  |  |  |  |  |
| *Kaiwarinus equula* |  |  |  |  |  |  |  |  |  |  |  |  |
| *Katsuwonus pelamis* |  |  |  |  |  | 213 |  |  |  |  |  | 35 |
| *Labracoglossa argenteiventris* |  |  |  |  |  |  |  |  | 211 | 154 |  |  |
| *Lagocephalus sceleratus* |  |  |  |  |  |  |  |  | 326 |  |  |  |
| *Lateolabrax japonicus* |  |  |  |  |  |  |  |  |  |  |  |  |
| *Lethrinus genivittatus* |  |  |  |  |  |  |  |  |  |  |  |  |
| *Lethrinus nebulosus* |  |  |  |  |  |  |  |  |  |  |  | 14 |
| *Macruronus novaezelandiae* |  |  |  |  |  |  |  |  |  |  |  |  |
| *Microcanthus strigatus* | 390 | 280 |  |  | 1,026 | 168 |  | 441 | 294 | 391 | 220 | 216 |
| *Microgadus proximus* |  |  |  |  |  |  |  |  |  |  |  |  |
| *Mugil cephalus* | 5,198 | 7,589 | 3,605 | 3,058 | 4,441 | 3,841 | 4,825 | 7,953 | 5,187 | 6,411 | 4,487 | 6,416 |
| *Mulloidichthys martinicus/Mulloidichthys vanicolensis/Mulloidichthys dentatus* |  |  |  |  |  |  |  |  |  | 179 | 215 | 30 |
| *Naso hexacanthus/Naso lituratus/Naso vlamingii/Naso brevirostris/Naso elegans* | 3,153 | 1,547 | 779 | 701 | 269 | 1,084 | 3,211 | 1,563 | 514 | 718 | 1,904 | 3,978 |
| *Naso unicornis/Naso tergus* | 1,014 |  |  |  |  |  |  |  |  |  |  | 53 |
| *Nuchequula nuchalis* |  |  |  |  |  |  |  |  |  | 239 |  |  |
| *Odonus niger* |  |  |  |  |  | 74 | 1,516 |  | 234 | 143 | 216 | 105 |
| *Omobranchus elegans* |  |  |  |  |  |  |  |  |  |  |  |  |
| *Oncorhynchus keta/Oncorhynchus kisutch/Oncorhynchus mykiss/Oncorhynchus masou ishikawae/Oncorhynchus masou masou/Oncorhynchus masou formosanus* |  |  |  | 39 |  |  | 785 | 3,204 | 101 | 55 |  | 16 |
| *Oplegnathus fasciatus* |  |  |  |  |  | 25 | 509 |  |  | 163 | 126 | 70 |
| *Oplegnathus punctatus* |  | 1,259 |  | 53 |  |  |  |  |  |  | 118 | 49 |
| *Ostorhinchus notatus* |  |  |  |  |  |  |  |  |  |  |  |  |
| *Ostracion rhinorhynchos* |  |  |  |  |  | 214 |  |  | 224 | 75 |  |  |
| *Ostracion meleagris meleagris* |  |  |  |  |  |  |  |  |  |  |  |  |
| *Pagrus major* | 1,458 |  |  |  | 266 | 215 | 629 |  |  |  | 203 | 75 |
| *Paracanthurus hepatus* |  |  |  |  |  |  |  |  |  |  |  |  |
| *Paracentropogon rubripinnis* |  |  |  |  |  |  |  |  |  |  |  |  |
| *Paracirrhites forsteri* |  |  |  |  |  |  |  |  |  | 141 |  |  |
| *Paramonacanthus otisensis/Paramonacanthus japonicus/Paramonacanthus choirocephalus* |  |  |  |  |  |  |  |  |  |  |  |  |
| *Parapristipoma trilineatum* |  |  |  |  |  |  |  |  |  |  |  |  |
| *Parupeneus multifasciatus* |  |  |  |  |  |  |  |  |  |  |  | 34 |
| *Petroscirtes breviceps* |  |  |  |  |  |  |  |  |  |  |  |  |
| *Pholis nebulosa* |  |  |  |  | 276 |  |  |  |  |  | 270 |  |
| *Platax teira/Platax orbicularis* |  |  |  |  |  |  |  |  |  |  |  |  |
| *Platichthys stellatus/Pleuronectes pinnifasciatus* |  |  |  |  |  |  |  |  |  |  |  |  |
| *Plectorhinchus cinctus* | 802 | 483 |  | 1,138 | 277 | 204 |  |  |  |  |  | 52 |
| *Plectroglyphidodon altus* |  |  |  |  |  | 65 |  |  |  |  |  |  |
| *Pleurogrammus azonus* |  |  |  |  |  |  | 1,147 | 2,909 |  |  |  |  |
| *Pleuronectes punctatissimus* |  |  |  |  |  |  |  |  |  |  |  |  |
| *Plotosus japonicus* | 1,074 | 1,655 | 777 | 964 | 1,704 | 3,250 | 84 | 459 | 300 | 541 | 960 | 423 |
| *Pollachius virens* |  |  |  |  |  |  |  |  |  |  | 82 |  |
| *Pomacanthus xanthometopon* |  |  |  |  |  |  |  |  |  |  |  |  |
| *Prionurus scalprum* |  |  |  |  |  |  | 32 |  |  |  |  | 81 |
| *Pseudanthias pleurotaenia* |  | 13 |  |  |  |  |  |  | 268 |  |  |  |
| *Pseudanthias rubrizonatus* |  | 40 | 47 |  | 129 | 52 |  |  |  | 15 |  |  |
| *Pseudanthias squamipinnis* | 2,917 | 1,816 | 2,336 | 1,218 | 1,005 | 1,538 | 1,949 | 2,032 | 1,352 | 2,007 | 1,592 | 1,229 |
| *Pterocaesio marri/Pterocaesio chrysozona* |  |  |  |  |  |  |  |  |  |  |  | 32 |
| *Rastrelliger kanagurta* |  |  |  |  |  |  |  |  |  |  |  |  |
| *Remora remora* |  | 1,199 |  |  |  | 279 |  | 72 |  | 257 |  | 122 |
| *Repomucenus valenciennei* |  |  |  |  |  |  |  |  |  |  |  |  |
| *Rhabdosargus sarba* | 267 |  |  |  |  | 225 |  |  |  |  |  |  |
| *Rhynchopelates oxyrhynchus* |  |  |  |  |  |  |  |  |  |  |  |  |
| *Rudarius ercodes* | 836 |  |  | 190 | 1,192 | 887 | 661 |  | 344 | 881 | 179 | 1,327 |
| *Sardinella albella* |  |  |  |  |  |  |  |  |  |  |  |  |
| *Sardinella longiceps* |  |  |  |  |  |  |  |  |  |  |  |  |
| *Sardinops melanosticta/Sardinops sagax* | 429 | 2,005 |  | 640 | 540 | 589 | 4,108 | 2,152 | 3,635 | 2,926 | 2,090 | 1,214 |
| *Scarus ghobban* |  | 72 |  | 33 | 120 |  |  |  |  | 12 | 77 | 21 |
| *Scarus ovifrons* |  |  |  |  |  |  |  |  |  |  |  |  |
| *Scomber australasicus/Scomber japonicus* | 1,020 |  |  | 986 | 1,694 | 501 |  | 1,092 | 1,752 | 673 | 791 | 246 |
| *Scorpaenopsis cirrosa* |  |  |  |  |  |  |  |  |  |  |  |  |
| *Sebastes miniatus/Sebastes taczanowskii/Sebastes steindachneri* |  |  |  |  |  |  |  |  |  |  |  |  |
| *Selenanthias analis* |  |  |  |  |  |  |  |  |  |  |  | 25 |
| *Semicossyphus reticulatus* |  |  |  |  | 169 | 222 | 424 |  | 385 | 221 |  | 54 |
| *Seriola quinqueradiata* |  |  |  | 139 |  |  |  |  | 48 |  |  |  |
| *Seriola dumerili* |  |  |  |  |  |  |  |  |  |  |  |  |
| *Seriola lalandi* |  |  |  |  |  |  |  |  |  |  |  |  |
| *Siganus virgatus/Siganus doliatus* |  |  |  |  | 260 | 168 |  |  |  |  | 141 |  |
| *Sillago japonica* |  |  |  |  |  |  |  |  |  |  |  |  |
| *Sphyrna lewini* |  |  |  |  |  |  |  |  |  |  |  |  |
| *Spratelloides gracilis* |  |  |  |  |  |  |  |  |  |  |  |  |
| *Stegastes fasciolatus/Stegastes altus* |  |  |  |  |  |  |  |  |  |  |  | 24 |
| *Stephanolepis cirrhifer* |  |  |  |  |  | 114 |  |  |  |  |  |  |
| *Stephanolepis setifer/Stephanolepis hispidus* |  |  |  |  |  |  |  |  |  |  |  |  |
| *Suezichthys gracilis* |  |  |  |  |  |  |  |  |  |  |  |  |
| *Syngnathus schlegeli* |  |  |  |  |  |  |  |  |  |  |  |  |
| *Thunnus maccoyii/Thunnus atlanticus/Thunnus obesus/Thunnus albacares* |  |  |  |  |  |  |  |  |  |  |  |  |
| *Trachurus mediterraneus/Trachurus symmetricus/Trachurus japonicus* |  |  |  |  |  |  |  |  | 436 | 71 |  | 77 |
| *Triacanthus biaculeatus* |  |  |  |  |  | 87 | 492 |  | 196 | 50 |  |  |
| *Tribolodon hakonensis* |  |  |  |  |  |  |  |  |  |  |  | 18 |
| *Upeneus japonicus* |  | 876 |  |  |  | 129 |  |  |  |  |  |  |

(B) Sterivex

| Species name | 0.5 L | | | | 1 L | | | | 2 L | | | |
| --- | --- | --- | --- | --- | --- | --- | --- | --- | --- | --- | --- | --- |
|  | 1 | 2 | 3 | 4 | 1 | 2 | 3 | 4 | 1 | 2 | 3 | 4 |
| Total read count | 28,590 | 25,513 | 28,311 | 25,539 | 24,378 | 28,936 | 29,356 | 28,067 | 27,340 | 23,348 | 61,189 | 61,611 |
| Total read count (after assignment to species) | 23,219 | 21,203 | 23,455 | 20,711 | 17,816 | 22,634 | 24,299 | 21,591 | 21,106 | 16,283 | 47,784 | 48,458 |
| *Acanthurus dussumieri* | 140 | 76 |  |  |  |  |  |  |  | 15 | 52 | 338 |
| *Acanthopagrus schlegelii/Acanthopagrus sivicolus* |  | 65 |  |  |  |  |  |  | 41 | 10 | 161 | 44 |
| *Acanthurus olivaceus* |  |  |  |  | 42 | 23 | 14 | 48 | 87 | 41 |  | 46 |
| *Acanthurus pyroferus* |  |  |  |  |  |  |  |  |  |  | 19 | 80 |
| *Acanthurus sohal* |  |  |  |  |  |  |  |  |  |  |  |  |
| *Alectis ciliaris* | 277 | 74 | 326 | 178 | 154 | 229 | 25 | 87 | 46 | 87 | 331 | 176 |
| *Alepes kleinii* |  |  |  |  |  |  |  |  |  |  |  |  |
| *Aluterus scriptus* | 750 |  |  | 112 |  | 21 | 17 | 158 | 11 |  | 262 | 384 |
| *Anguilla japonica* |  |  |  |  |  |  |  |  |  |  | 62 |  |
| *Antennarius commerson* |  | 43 | 25 |  |  | 22 |  | 21 | 22 |  | 152 | 66 |
| *Apogonichthyoides niger* |  |  |  |  |  |  |  |  |  |  |  |  |
| *Arothron hispidus* |  |  |  |  |  | 20 |  |  |  |  |  | 81 |
| *Auxis rochei* |  |  |  |  |  |  |  |  |  |  |  |  |
| *Caesio cuning* | 89 | 117 | 165 |  | 140 | 344 |  | 198 | 28 | 35 | 17 | 137 |
| *Carangoides equula* |  |  |  |  |  |  |  |  |  |  |  |  |
| *Centropyge bicolor* |  |  |  |  |  |  | 26 |  |  |  |  |  |
| *Chaetodon auripes/Chaetodon adiergastos/Chaetodon fasciatus* |  | 44 | 147 | 91 | 46 |  |  | 24 | 44 | 15 | 47 | 55 |
| *Chaetodon ephippium* |  |  |  |  |  |  |  |  |  |  |  |  |
| *Chaetodon kleinii* |  |  |  |  |  |  |  |  |  |  | 25 |  |
| *Chaetodon lunula* |  |  |  |  |  |  |  | 140 | 40 |  | 15 | 48 |
| *Chaetodon melannotus* |  |  |  |  |  |  |  |  |  |  |  | 13 |
| *Cheilodactylus quadricornis/Cheilodactylus zonatus* |  |  |  |  |  |  |  |  |  |  |  |  |
| *Cheilodactylus zebra/Cheilodactylus quadricornis* | 142 | 133 | 284 | 391 | 82 | 90 | 38 | 1,403 | 255 | 155 | 509 | 237 |
| *Chilomycterus reticulatus* |  |  |  |  |  |  |  |  |  |  |  |  |
| *Cirrhitichthys aureus* | 171 | 175 | 22 |  | 141 | 21 |  | 105 | 77 | 91 | 115 | 103 |
| *Clupea pallasii* |  | 55 | 171 |  | 30 | 56 |  | 137 | 41 | 12 | 127 |  |
| *Cololabis saira* |  |  |  |  |  |  |  |  |  |  |  |  |
| *Coris aygula* | 153 | 81 |  | 253 | 44 | 203 | 1,947 | 250 | 155 | 289 | 106 | 451 |
| *Cromileptes altivelis* |  |  |  |  | 14 |  |  |  |  |  |  |  |
| *Ctenochaetus tominiensis* |  |  |  |  |  |  |  |  |  |  |  |  |
| *Decapterus russelli/Decapterus maruadsi* |  |  |  |  |  |  |  |  |  |  | 35 |  |
| *Dictyosoma burgeri* |  |  | 130 |  | 99 |  | 14 | 148 | 14 | 26 | 111 | 183 |
| *Diodon holocanthus/Diodon liturosus* |  |  |  |  |  | 76 |  |  |  |  | 62 |  |
| *Diodon hystrix* | 1,738 | 1,278 | 2,252 | 436 | 1,003 | 549 | 371 | 1,319 | 810 | 347 | 2,413 | 2,521 |
| *Ditrema temminckii temminckii/Ditrema viride* | 314 | 350 | 343 | 266 | 600 | 101 | 82 | 141 | 460 | 236 | 1,526 | 620 |
| *Echeneis naucrates* |  |  |  |  |  |  |  |  | 38 |  |  |  |
| *Eleginus gracilis* |  |  |  |  |  |  |  | 23 |  |  |  |  |
| *Engraulis ringens* |  |  |  |  |  |  |  |  |  |  |  |  |
| *Enophrys diceraus* |  |  |  |  |  |  |  |  |  |  |  |  |
| *Epinephelus akaara* | 216 | 170 | 871 | 712 | 412 | 281 | 240 | 1,090 | 852 | 985 | 1,525 | 2,023 |
| *Epinephelus areolatus* | 142 | 82 | 178 | 278 | 147 | 71 |  | 34 | 64 | 39 | 235 | 268 |
| *Epinephelus awoara/Epinephelus coioides* | 146 | 185 | 170 | 517 | 176 | 46 | 28 | 193 | 307 | 230 | 699 | 571 |
| *Epinephelus coioides/Epinephelus malabaricus* | 181 |  | 463 | 159 | 71 | 19 | 20 | 110 | 105 | 81 | 138 | 186 |
| *Epinephelus fasciatus* |  | 42 |  |  | 499 | 54 |  | 116 | 87 | 171 | 28 | 418 |
| *Epinephelus morrhua* |  |  |  |  |  |  |  |  |  |  |  |  |
| *Epinephelus bruneus* | 173 | 160 | 144 |  |  | 135 | 15 |  | 64 | 129 |  | 187 |
| *Epinephelus octofasciatus* |  |  | 196 |  | 204 | 70 | 23 | 138 | 115 | 45 | 334 | 322 |
| *Epinephelus septemfasciatus* |  |  |  |  |  | 30 |  |  | 19 |  | 112 |  |
| *Epinephelus trimaculatus* | 111 | 214 | 64 |  | 28 |  |  | 102 | 13 |  | 109 | 74 |
| *Euthynnus alletteratus* |  |  |  |  |  |  |  |  |  |  |  |  |
| *Gadus chalcogrammus/Gadus morhua/Gadus finnmarchicus* | 103 | 83 | 505 | 26 | 392 | 167 | 69 | 97 | 21 | 41 | 142 | 50 |
| *Gerres oyena/Gerres equulus* |  |  | 82 |  | 39 |  | 39 | 83 | 39 | 55 | 28 | 100 |
| *Girella leonina/Girella melanichthys* |  |  |  |  |  |  |  | 48 | 55 |  | 175 |  |
| *Girella punctata* |  |  | 156 |  |  |  |  |  |  |  |  |  |
| *Gymnocanthus herzensteini* |  |  |  |  |  |  |  |  |  |  |  |  |
| *Gymnocranius griseus* |  |  |  |  |  |  |  |  |  |  |  |  |
| *Gymnogobius petschiliensis/Gymnogobius opperiens* |  |  |  |  |  | 10 |  |  |  |  |  |  |
| *Gymnomuraena zebra* |  |  |  |  |  |  |  |  | 94 |  |  |  |
| *Gymnothorax kidako* | 3,677 | 4,580 | 3,716 | 3,645 | 2,750 | 2,517 | 1,172 | 2,653 | 4,087 | 1,455 | 7,540 | 7,568 |
| *Gymnothorax reticularis/Gymnothorax minor* |  |  |  |  |  | 23 |  |  | 35 |  |  | 129 |
| *Gymnothorax flavimarginatus* |  |  | 97 |  |  |  |  |  |  |  |  |  |
| *Halichoeres tenuispinis* | 135 |  |  |  |  |  |  |  |  |  |  |  |
| *Hapalogenys sennin* |  | 43 |  |  |  |  |  |  |  | 20 | 54 | 149 |
| *Hemitaurichthys polylepis/Hemitaurichthys zoster* |  |  |  |  | 41 | 48 |  |  | 53 | 29 | 52 |  |
| *Hemitripterus villosus* |  |  |  |  |  |  |  | 47 |  |  |  |  |
| *Heterodontus zebra/Heterodontus japonicus* |  |  |  |  |  |  |  |  |  |  |  |  |
| *Hyporthodus septemfasciatus* |  |  |  |  |  |  |  |  |  |  |  |  |
| *Inimicus japonicus* |  |  |  |  |  |  |  |  |  |  | 142 | 100 |
| *Istigobius campbelli* |  |  |  |  |  |  |  |  |  |  |  |  |
| *Kaiwarinus equula* |  |  |  |  |  |  |  |  |  |  |  | 86 |
| *Katsuwonus pelamis* |  |  |  |  |  |  |  |  |  |  |  |  |
| *Labracoglossa argenteiventris* | 98 |  |  | 226 | 20 |  |  |  | 37 |  |  | 99 |
| *Lagocephalus sceleratus* |  |  |  |  |  |  |  |  |  | 27 |  | 32 |
| *Lateolabrax japonicus* |  |  |  |  |  | 111 |  |  | 30 | 20 | 26 |  |
| *Lethrinus genivittatus* |  |  |  |  |  | 43 |  |  |  |  |  |  |
| *Lethrinus nebulosus* |  |  |  |  |  |  | 42 |  |  |  | 69 |  |
| *Macruronus novaezelandiae* |  |  |  |  |  |  |  |  | 19 |  |  |  |
| *Microcanthus strigatus* | 568 | 62 | 568 | 340 | 621 | 17 | 87 | 546 | 879 | 553 | 1,638 | 1,979 |
| *Microgadus proximus* |  |  |  |  |  |  |  | 23 |  |  |  |  |
| *Mugil cephalus* | 6,480 | 2,844 | 3,539 | 4,010 | 3,207 | 3,189 | 937 | 3,668 | 4,135 | 4,832 | 10,446 | 10,177 |
| *Mulloidichthys martinicus/Mulloidichthys vanicolensis/Mulloidichthys dentatus* |  |  |  |  |  | 26 |  | 20 |  |  |  | 45 |
| *Naso hexacanthus/Naso lituratus/Naso vlamingii/Naso brevirostris/Naso elegans* | 603 | 4,493 | 2,117 | 1,192 | 892 | 8,597 | 18,040 | 2,956 | 678 | 376 | 1,371 | 918 |
| *Naso unicornis/Naso tergus* |  |  |  |  |  | 73 |  | 208 | 16 | 46 | 80 | 66 |
| *Nuchequula nuchalis* |  |  | 410 |  |  |  |  | 27 |  |  | 28 | 83 |
| *Odonus niger* | 160 | 222 |  | 189 | 87 | 19 |  |  |  | 74 | 27 | 43 |
| *Omobranchus elegans* |  |  |  |  | 11 |  |  |  |  |  |  |  |
| *Oncorhynchus keta/Oncorhynchus kisutch/Oncorhynchus mykiss/Oncorhynchus masou ishikawae/Oncorhynchus masou masou/Oncorhynchus masou formosanus* |  | 76 |  |  | 28 | 18 | 23 | 224 | 105 | 21 | 41 | 45 |
| *Oplegnathus fasciatus* | 80 | 69 |  | 68 | 96 |  | 10 | 35 | 18 | 108 | 333 | 277 |
| *Oplegnathus punctatus* |  | 80 | 216 | 118 | 131 | 43 | 32 | 49 | 127 | 110 | 233 | 472 |
| *Ostorhinchus notatus* |  |  |  |  |  |  |  |  | 40 | 39 |  |  |
| *Ostracion rhinorhynchos* |  |  |  |  |  | 34 |  |  | 19 | 66 | 139 |  |
| *Ostracion meleagris meleagris* |  |  |  |  |  |  |  |  |  |  |  |  |
| *Pagrus major* | 60 | 227 | 566 |  | 285 | 218 | 57 | 145 | 92 | 117 | 400 | 426 |
| *Paracanthurus hepatus* |  |  |  |  |  |  |  |  |  |  |  |  |
| *Paracentropogon rubripinnis* |  | 74 |  |  |  |  |  |  | 15 |  |  |  |
| *Paracirrhites forsteri* |  |  |  |  |  |  |  |  |  |  |  |  |
| *Paramonacanthus otisensis/Paramonacanthus japonicus/Paramonacanthus choirocephalus* |  |  |  |  |  |  |  |  |  |  | 37 |  |
| *Parapristipoma trilineatum* |  |  |  |  | 26 |  |  |  | 37 |  |  | 283 |
| *Parupeneus multifasciatus* |  |  |  |  |  |  |  |  |  |  |  |  |
| *Petroscirtes breviceps* |  |  |  |  |  |  |  |  |  |  |  | 41 |
| *Pholis nebulosa* |  |  |  |  |  |  |  |  |  |  |  |  |
| *Platax teira/Platax orbicularis* |  |  |  |  |  |  |  |  |  |  |  |  |
| *Platichthys stellatus/Pleuronectes pinnifasciatus* |  |  |  |  |  |  |  |  |  |  |  |  |
| *Plectorhinchus cinctus* | 2,262 | 1,577 | 1,510 | 720 | 249 | 895 | 63 | 755 | 951 | 118 | 1,426 | 3,172 |
| *Plectroglyphidodon altus* |  |  |  |  |  |  |  |  |  |  |  |  |
| *Pleurogrammus azonus* |  |  |  |  |  |  |  | 25 |  | 47 |  |  |
| *Pleuronectes punctatissimus* |  |  |  |  |  |  |  |  |  |  |  |  |
| *Plotosus japonicus* | 445 | 322 | 157 | 1,187 | 790 | 651 | 127 |  | 563 | 897 | 3,916 | 2,331 |
| *Pollachius virens* |  |  |  |  |  |  |  |  |  |  | 44 |  |
| *Pomacanthus xanthometopon* |  |  |  |  |  |  |  | 64 | 34 |  |  |  |
| *Prionurus scalprum* |  |  |  | 162 | 136 | 17 |  | 49 | 69 | 83 |  | 74 |
| *Pseudanthias pleurotaenia* | 60 |  |  |  | 43 |  |  | 97 | 42 |  | 149 | 84 |
| *Pseudanthias rubrizonatus* |  |  | 60 | 86 | 13 | 50 | 15 | 23 | 12 | 12 | 17 | 17 |
| *Pseudanthias squamipinnis* | 1,449 | 1,070 | 1,864 | 2,061 | 889 | 1,656 | 207 | 1,041 | 971 | 1,046 | 2,485 | 2,074 |
| *Pterocaesio marri/Pterocaesio chrysozona* |  |  |  | 56 | 55 |  |  |  |  | 32 |  |  |
| *Rastrelliger kanagurta* |  |  |  |  |  |  |  |  |  |  |  |  |
| *Remora remora* |  | 111 | 63 | 1,066 | 58 | 233 |  | 129 | 57 | 109 | 187 | 254 |
| *Repomucenus valenciennei* |  |  |  |  |  |  |  |  |  |  |  |  |
| *Rhabdosargus sarba* |  | 91 |  |  | 10 | 105 | 23 | 88 | 55 | 118 | 437 | 314 |
| *Rhynchopelates oxyrhynchus* | 77 |  |  | 215 |  |  |  | 62 |  |  |  |  |
| *Rudarius ercodes* | 555 | 306 | 487 |  | 269 | 601 | 132 | 361 | 540 | 291 | 1,902 | 1,487 |
| *Sardinella albella* |  |  |  |  |  |  |  |  |  |  |  |  |
| *Sardinella longiceps* |  |  |  |  |  |  |  |  |  | 13 |  |  |
| *Sardinops melanosticta/Sardinops sagax* | 881 | 742 | 942 | 459 | 1,319 | 347 | 252 | 1,156 | 1,384 | 1,367 | 2,604 | 3,251 |
| *Scarus ghobban* | 51 | 113 | 10 | 86 | 44 | 10 |  | 53 | 1,191 | 65 | 288 | 313 |
| *Scarus ovifrons* |  |  |  |  |  |  |  |  |  |  |  | 26 |
| *Scomber australasicus/Scomber japonicus* | 382 | 342 | 95 | 1,194 | 993 | 282 | 80 | 287 | 327 | 454 | 1,242 | 1,797 |
| *Scorpaenopsis cirrosa* |  |  |  |  |  |  |  |  | 22 |  |  |  |
| *Sebastes miniatus/Sebastes taczanowskii/Sebastes steindachneri* |  |  |  |  |  |  |  | 42 |  |  |  |  |
| *Selenanthias analis* |  |  |  |  |  |  |  |  |  |  |  |  |
| *Semicossyphus reticulatus* |  |  |  | 190 | 63 |  |  | 47 | 243 | 160 | 310 | 244 |
| *Seriola quinqueradiata* |  |  |  |  | 12 |  |  |  |  |  |  |  |
| *Seriola dumerili* |  |  |  |  |  |  |  |  |  |  |  |  |
| *Seriola lalandi* |  |  |  |  | 18 |  |  |  |  |  |  |  |
| *Siganus virgatus/Siganus doliatus* |  |  |  |  |  | 39 |  |  | 35 | 44 |  | 21 |
| *Sillago japonica* |  |  |  |  | 41 |  |  |  |  |  |  |  |
| *Sphyrna lewini* |  |  |  |  |  |  |  |  |  |  |  |  |
| *Spratelloides gracilis* |  |  |  |  |  |  |  | 67 |  | 13 |  |  |
| *Stegastes fasciolatus/Stegastes altus* | 180 |  |  |  |  |  |  | 108 |  |  |  |  |
| *Stephanolepis cirrhifer* | 170 | 74 | 109 |  | 23 | 21 |  |  | 66 | 40 | 32 | 29 |
| *Stephanolepis setifer/Stephanolepis hispidus* |  |  |  |  |  |  |  |  |  |  |  |  |
| *Suezichthys gracilis* |  |  |  |  |  |  |  |  |  |  |  |  |
| *Syngnathus schlegeli* |  |  |  |  |  |  |  |  |  |  |  |  |
| *Thunnus maccoyii/Thunnus atlanticus/Thunnus obesus/Thunnus albacares* |  |  |  |  |  |  |  | 36 |  |  | 67 |  |
| *Trachurus mediterraneus/Trachurus symmetricus/Trachurus japonicus* |  | 212 |  | 22 | 223 | 32 | 32 | 157 | 43 | 255 | 149 | 123 |
| *Triacanthus biaculeatus* |  |  | 235 |  | 10 | 43 |  |  | 11 | 57 | 61 | 47 |
| *Tribolodon hakonensis* |  |  |  |  |  |  |  |  |  |  |  |  |
| *Upeneus japonicus* |  | 46 |  |  |  | 38 |  | 130 | 64 | 34 | 140 |  |

(C) QuickConc Vacuum

| Species name | 0.5 L | | | | 1 L | | | | 2 L | | | |
| --- | --- | --- | --- | --- | --- | --- | --- | --- | --- | --- | --- | --- |
|  | 1 | 2 | 3 | 4 | 1 | 2 | 3 | 4 | 1 | 2 | 3 | 4 |
| Total read count | 70,053 | 55,279 | 36,793 | 46,345 | 47,157 | 60,585 | 51,295 | 45,101 | 65,397 | 54,066 | 54,420 | 55,642 |
| Total read count (after assignment to species) | 56,367 | 45,717 | 31,219 | 39,622 | 38,778 | 45,425 | 39,059 | 36,442 | 50,887 | 53,144 | 42,372 | 42,337 |
| *Acanthurus dussumieri* |  |  |  |  |  |  | 50 |  | 141 | 24 |  | 40 |
| *Acanthopagrus schlegelii/Acanthopagrus sivicolus* |  |  |  |  |  | 100 |  |  |  |  | 25 | 38 |
| *Acanthurus olivaceus* | 282 | 91 | 114 |  | 75 |  |  | 55 |  | 57 | 10 | 21 |
| *Acanthurus pyroferus* |  |  |  |  |  |  |  |  |  |  |  | 15 |
| *Acanthurus sohal* |  |  |  |  |  |  |  |  |  | 57 |  | 21 |
| *Alectis ciliaris* | 787 | 1,096 | 240 | 682 | 256 | 203 | 271 | 157 | 77 | 198 | 57 | 178 |
| *Alepes kleinii* |  |  |  |  |  |  |  |  |  | 17 |  |  |
| *Aluterus scriptus* |  |  | 157 | 88 |  | 140 | 126 | 112 | 239 | 61 | 107 | 206 |
| *Anguilla japonica* |  |  |  |  |  |  |  |  |  |  |  |  |
| *Antennarius commerson* |  |  |  | 68 |  | 57 | 120 | 91 |  |  | 39 |  |
| *Apogonichthyoides niger* |  |  |  |  |  |  | 303 |  |  |  | 42 |  |
| *Arothron hispidus* |  |  |  |  |  |  |  |  |  |  |  |  |
| *Auxis rochei* |  |  |  |  |  |  |  |  | 297 |  |  |  |
| *Caesio cuning* |  | 495 |  | 105 | 285 | 110 | 303 | 40 | 166 | 35 | 80 | 80 |
| *Carangoides equula* |  |  |  |  |  |  |  |  |  |  |  |  |
| *Centropyge bicolor* |  |  |  | 155 |  |  | 323 |  | 62 |  | 18 |  |
| *Chaetodon auripes/Chaetodon adiergastos/Chaetodon fasciatus* |  |  |  |  | 49 | 69 |  |  | 32 | 57 | 39 | 33 |
| *Chaetodon ephippium* |  |  |  |  |  |  |  |  |  |  |  |  |
| *Chaetodon kleinii* |  |  |  |  |  |  |  |  |  |  | 13 | 17 |
| *Chaetodon lunula* |  |  | 64 |  |  |  |  | 89 |  |  |  |  |
| *Chaetodon melannotus* |  |  |  |  |  |  |  |  |  |  |  |  |
| *Cheilodactylus quadricornis/Cheilodactylus zonatus* |  |  |  |  |  |  |  |  |  |  |  |  |
| *Cheilodactylus zebra/Cheilodactylus quadricornis* | 236 | 255 | 282 | 125 | 78 | 70 | 91 | 150 | 268 | 139 | 427 | 182 |
| *Chilomycterus reticulatus* |  |  |  |  |  |  |  |  |  |  |  |  |
| *Cirrhitichthys aureus* | 501 | 521 | 427 | 196 | 442 | 103 |  | 88 | 144 | 72 | 62 | 167 |
| *Clupea pallasii* |  | 796 |  |  | 69 | 249 | 664 | 421 | 176 | 341 | 127 | 85 |
| *Cololabis saira* |  |  |  |  |  |  |  |  |  | 500 |  | 13 |
| *Coris aygula* | 1,070 | 788 | 1,381 | 986 | 581 | 2,550 | 568 | 771 | 1,093 | 524 | 624 | 546 |
| *Cromileptes altivelis* |  |  |  |  |  |  |  |  |  |  |  |  |
| *Ctenochaetus tominiensis* |  |  |  |  |  |  |  |  |  |  |  |  |
| *Decapterus russelli/Decapterus maruadsi* |  |  |  |  |  |  | 103 | 26 |  |  |  |  |
| *Dictyosoma burgeri* |  | 88 | 443 |  | 169 | 411 | 43 |  | 170 | 71 | 44 | 42 |
| *Diodon holocanthus/Diodon liturosus* |  |  |  |  |  |  |  |  |  |  |  |  |
| *Diodon hystrix* | 4,577 | 2,779 | 2,648 | 1,486 | 1,111 | 5,191 | 4,046 | 6,028 | 4,455 | 4,433 | 2,841 | 3,649 |
| *Ditrema temminckii temminckii/Ditrema viride* | 1,185 | 1,039 | 255 | 261 | 490 | 310 | 259 | 178 | 197 | 219 | 211 | 968 |
| *Echeneis naucrates* |  |  |  |  |  |  |  |  |  |  |  |  |
| *Eleginus gracilis* |  |  |  |  |  |  |  |  |  |  |  |  |
| *Engraulis ringens* |  |  |  |  |  |  |  |  |  |  |  |  |
| *Enophrys diceraus* |  |  |  |  |  |  |  |  | 17 |  |  |  |
| *Epinephelus akaara* | 858 | 366 | 226 | 222 | 296 | 546 | 343 | 178 | 471 | 230 | 610 | 860 |
| *Epinephelus areolatus* |  | 448 | 562 |  | 16 | 143 |  |  | 317 | 77 | 119 | 145 |
| *Epinephelus awoara/Epinephelus coioides* |  |  | 172 | 173 | 55 | 150 | 402 | 260 | 207 | 63 | 151 | 393 |
| *Epinephelus coioides/Epinephelus malabaricus* |  | 349 | 647 |  | 412 | 147 | 148 |  | 69 | 23 | 40 | 59 |
| *Epinephelus fasciatus* | 504 |  |  | 131 | 219 | 290 | 115 | 367 | 127 | 19 | 130 | 294 |
| *Epinephelus morrhua* |  |  |  |  |  |  |  |  |  |  |  |  |
| *Epinephelus bruneus* |  |  |  |  |  |  |  |  | 58 |  | 63 |  |
| *Epinephelus octofasciatus* | 502 |  |  | 18 | 44 |  | 187 | 31 | 36 | 23 | 89 | 153 |
| *Epinephelus septemfasciatus* |  | 12 |  | 88 |  |  | 55 | 85 |  |  | 27 | 70 |
| *Epinephelus trimaculatus* |  |  |  |  |  |  |  | 315 |  | 69 |  |  |
| *Euthynnus alletteratus* |  |  |  |  |  |  |  |  |  | 12 |  |  |
| *Gadus chalcogrammus/Gadus morhua/Gadus finnmarchicus* | 622 | 643 | 348 | 129 | 1,269 | 1,556 | 1,733 | 1,154 | 3,792 | 12,351 | 1,464 | 1,131 |
| *Gerres oyena/Gerres equulus* | 224 |  | 94 |  | 413 | 61 |  | 17 | 79 |  | 15 | 35 |
| *Girella leonina/Girella melanichthys* |  | 181 |  |  |  | 95 |  |  | 15 | 19 |  | 23 |
| *Girella punctata* |  | 131 |  |  |  |  | 113 |  |  | 10 |  |  |
| *Gymnocanthus herzensteini* | 308 |  |  |  |  |  |  |  |  | 13 |  |  |
| *Gymnocranius griseus* |  |  |  |  |  |  | 10 |  |  |  |  |  |
| *Gymnogobius petschiliensis/Gymnogobius opperiens* |  |  |  |  |  |  |  |  |  |  |  |  |
| *Gymnomuraena zebra* |  |  |  |  |  | 185 |  |  |  | 36 |  | 30 |
| *Gymnothorax kidako* | 6,654 | 4,941 | 2,988 | 5,453 | 2,954 | 6,394 | 6,561 | 6,488 | 5,617 | 4,464 | 8,222 | 7,839 |
| *Gymnothorax reticularis/Gymnothorax minor* |  |  |  | 168 |  | 62 |  | 56 |  | 12 | 61 | 95 |
| *Gymnothorax flavimarginatus* |  |  |  |  |  |  |  |  | 87 | 18 |  |  |
| *Halichoeres tenuispinis* |  |  |  |  |  |  |  |  |  |  |  |  |
| *Hapalogenys sennin* | 606 | 138 |  |  |  |  |  | 24 |  | 37 | 31 |  |
| *Hemitaurichthys polylepis/Hemitaurichthys zoster* | 705 | 490 |  |  | 32 |  | 169 |  | 46 | 44 | 47 | 104 |
| *Hemitripterus villosus* |  |  |  |  |  |  |  |  |  |  |  |  |
| *Heterodontus zebra/Heterodontus japonicus* |  |  |  |  |  |  | 13 |  |  |  |  |  |
| *Hyporthodus septemfasciatus* |  |  |  |  |  |  |  |  |  |  |  |  |
| *Inimicus japonicus* |  |  |  |  | 73 |  |  | 22 |  |  | 24 |  |
| *Istigobius campbelli* |  |  |  |  |  | 72 |  |  |  |  |  |  |
| *Kaiwarinus equula* |  |  |  |  |  |  |  |  |  | 11 |  |  |
| *Katsuwonus pelamis* |  |  |  |  |  |  |  |  | 50 | 74 | 23 | 38 |
| *Labracoglossa argenteiventris* |  |  |  | 213 | 50 |  | 74 |  | 45 |  | 14 | 37 |
| *Lagocephalus sceleratus* |  |  |  |  |  |  |  |  |  |  | 17 |  |
| *Lateolabrax japonicus* |  |  |  |  |  |  |  |  |  | 14 | 20 | 15 |
| *Lethrinus genivittatus* |  | 194 |  |  |  |  |  |  |  |  | 12 |  |
| *Lethrinus nebulosus* |  |  |  |  |  |  |  |  | 67 |  |  | 14 |
| *Macruronus novaezelandiae* |  |  |  |  |  |  |  |  |  |  |  |  |
| *Microcanthus strigatus* | 3,650 | 1,142 | 900 | 743 | 691 | 352 | 966 | 1,653 | 721 | 698 | 1,282 | 1,339 |
| *Microgadus proximus* |  |  |  |  |  |  |  |  |  |  |  |  |
| *Mugil cephalus* | 12,808 | 5,607 | 6,264 | 4,338 | 5,878 | 5,808 | 7,697 | 3,288 | 4,144 | 2,955 | 5,221 | 5,837 |
| *Mulloidichthys martinicus/Mulloidichthys vanicolensis/Mulloidichthys dentatus* |  | 247 |  |  |  | 24 |  |  |  | 31 |  | 27 |
| *Naso hexacanthus/Naso lituratus/Naso vlamingii/Naso brevirostris/Naso elegans* | 919 | 1,090 | 1,754 | 63 | 8,876 | 1,296 | 218 | 1,028 | 1,765 | 592 | 1,423 | 1,164 |
| *Naso unicornis/Naso tergus* |  | 78 |  | 138 |  | 75 |  |  | 42 | 37 | 103 | 163 |
| *Nuchequula nuchalis* |  |  |  |  | 48 |  | 50 |  | 49 |  |  | 28 |
| *Odonus niger* |  | 273 |  |  | 71 | 232 | 214 | 251 | 129 | 93 | 164 | 95 |
| *Omobranchus elegans* |  |  |  |  |  |  |  |  |  |  |  |  |
| *Oncorhynchus keta/Oncorhynchus kisutch/Oncorhynchus mykiss/Oncorhynchus masou ishikawae/Oncorhynchus masou masou/Oncorhynchus masou formosanus* |  |  |  |  |  | 295 |  | 140 | 510 | 136 | 112 | 98 |
| *Oplegnathus fasciatus* |  | 400 | 132 |  |  | 130 | 68 |  | 39 | 75 | 126 | 56 |
| *Oplegnathus punctatus* | 513 | 100 | 444 |  | 115 | 167 | 86 | 86 | 166 | 30 | 237 | 243 |
| *Ostorhinchus notatus* |  |  |  |  |  |  |  |  |  |  |  |  |
| *Ostracion rhinorhynchos* |  |  | 88 |  | 40 |  |  |  | 14 |  |  | 30 |
| *Ostracion meleagris meleagris* |  |  |  |  |  |  |  |  |  |  |  | 30 |
| *Pagrus major* |  | 75 |  | 171 | 101 | 94 | 68 | 203 | 308 | 280 | 323 | 340 |
| *Paracanthurus hepatus* |  |  |  |  |  |  |  | 152 |  |  |  |  |
| *Paracentropogon rubripinnis* |  |  |  |  |  |  |  |  |  |  |  |  |
| *Paracirrhites forsteri* |  |  |  |  |  |  |  |  |  |  |  |  |
| *Paramonacanthus otisensis/Paramonacanthus japonicus/Paramonacanthus choirocephalus* |  |  |  |  |  |  |  |  |  | 11 |  |  |
| *Parapristipoma trilineatum* | 420 |  |  | 131 | 63 | 63 | 54 | 30 |  | 23 | 44 | 19 |
| *Parupeneus multifasciatus* |  |  |  |  |  | 45 |  | 44 |  |  |  |  |
| *Petroscirtes breviceps* |  |  | 153 |  |  |  | 37 |  |  |  |  |  |
| *Pholis nebulosa* |  |  |  |  |  |  |  |  |  |  |  |  |
| *Platax teira/Platax orbicularis* |  | 142 |  |  |  |  |  |  |  |  |  |  |
| *Platichthys stellatus/Pleuronectes pinnifasciatus* |  |  |  |  |  |  |  |  | 17 |  |  |  |
| *Plectorhinchus cinctus* |  | 138 |  |  |  | 4,506 | 278 | 1,117 | 6,150 | 1,291 | 2,453 | 2,615 |
| *Plectroglyphidodon altus* |  |  |  |  |  |  |  |  |  |  |  |  |
| *Pleurogrammus azonus* | 84 | 101 |  |  | 24 | 516 | 81 | 87 | 71 | 62 | 68 | 12 |
| *Pleuronectes punctatissimus* |  |  |  |  |  |  |  |  | 16 |  |  |  |
| *Plotosus japonicus* | 1,356 | 850 | 1,103 | 426 | 536 | 1,462 | 1,022 | 1,712 | 5,578 | 886 | 1,359 | 4,770 |
| *Pollachius virens* |  |  |  |  |  |  |  |  |  |  |  |  |
| *Pomacanthus xanthometopon* |  |  |  |  |  |  | 31 |  |  |  | 17 |  |
| *Prionurus scalprum* | 431 | 282 |  |  |  | 101 |  |  | 12 | 30 |  | 10 |
| *Pseudanthias pleurotaenia* |  | 480 |  | 82 | 40 |  | 19 |  |  | 21 |  |  |
| *Pseudanthias rubrizonatus* |  |  | 83 | 44 |  | 105 | 26 | 30 | 11 |  | 23 |  |
| *Pseudanthias squamipinnis* | 3,329 | 2,449 | 1,479 | 1,262 | 1,531 | 2,953 | 2,840 | 1,075 | 1,373 | 1,222 | 1,247 | 1,345 |
| *Pterocaesio marri/Pterocaesio chrysozona* | 52 |  |  |  |  | 399 | 44 |  | 90 | 43 |  | 25 |
| *Rastrelliger kanagurta* |  |  |  |  |  |  |  |  | 15 |  |  |  |
| *Remora remora* |  |  |  |  |  | 235 | 54 | 131 | 57 | 27 | 128 | 107 |
| *Repomucenus valenciennei* | 154 |  |  |  |  |  |  |  |  |  |  | 12 |
| *Rhabdosargus sarba* |  |  | 286 |  |  |  |  |  | 145 | 52 | 82 | 101 |
| *Rhynchopelates oxyrhynchus* |  |  |  |  |  |  |  |  |  | 15 |  |  |
| *Rudarius ercodes* | 4,065 | 1,227 | 1,591 | 238 | 853 | 819 | 1,235 | 396 | 542 | 623 | 701 | 665 |
| *Sardinella albella* |  |  |  |  |  |  |  |  |  | 10 |  |  |
| *Sardinella longiceps* | 3,261 |  |  |  |  |  |  | 28 | 47 |  |  |  |
| *Sardinops melanosticta/Sardinops sagax* | 3,375 | 14,472 | 2,682 | 20,638 | 9,245 | 4,920 | 3,961 | 6,493 | 9,169 | 7,600 | 10,488 | 2,922 |
| *Scarus ghobban* |  | 51 | 19 | 122 | 10 | 45 |  | 22 | 125 | 15 | 30 | 132 |
| *Scarus ovifrons* |  |  |  |  |  | 42 |  |  | 11 |  |  |  |
| *Scomber australasicus/Scomber japonicus* | 1,571 |  | 600 | 57 | 882 | 733 | 1,837 | 437 | 295 | 332 | 375 | 1,162 |
| *Scorpaenopsis cirrosa* |  |  |  |  |  |  |  | 59 |  |  |  |  |
| *Sebastes miniatus/Sebastes taczanowskii/Sebastes steindachneri* |  |  |  |  |  |  |  |  |  |  |  |  |
| *Selenanthias analis* |  |  |  |  |  |  |  |  |  |  |  |  |
| *Semicossyphus reticulatus* |  | 357 | 1,774 |  | 23 | 160 | 464 | 381 | 55 | 33 | 45 | 310 |
| *Seriola quinqueradiata* |  |  |  |  | 26 | 13 | 18 |  |  | 13 | 13 |  |
| *Seriola dumerili* |  |  |  |  |  |  |  |  |  |  |  | 10 |
| *Seriola lalandi* |  |  |  |  |  |  |  |  |  |  |  |  |
| *Siganus virgatus/Siganus doliatus* | 575 |  |  |  |  |  |  | 133 | 30 | 14 | 83 | 53 |
| *Sillago japonica* |  |  |  |  |  |  |  |  | 33 | 23 |  |  |
| *Sphyrna lewini* |  |  | 23 |  |  |  |  |  |  |  |  |  |
| *Spratelloides gracilis* |  |  |  |  |  |  |  |  |  | 11,187 |  |  |
| *Stegastes fasciolatus/Stegastes altus* |  |  | 399 |  |  |  |  |  |  | 12 | 21 | 15 |
| *Stephanolepis cirrhifer* |  |  |  |  | 60 |  | 48 | 58 | 89 |  | 41 | 66 |
| *Stephanolepis setifer/Stephanolepis hispidus* |  |  |  |  | 60 |  |  |  |  |  |  |  |
| *Suezichthys gracilis* |  |  |  |  |  |  |  |  |  | 21 |  |  |
| *Syngnathus schlegeli* |  |  |  | 57 |  |  |  |  |  |  |  |  |
| *Thunnus maccoyii/Thunnus atlanticus/Thunnus obesus/Thunnus albacares* |  |  |  | 180 |  | 43 | 94 |  | 136 |  |  | 17 |
| *Trachurus mediterraneus/Trachurus symmetricus/Trachurus japonicus* |  | 315 | 293 | 185 | 167 | 182 | 316 | 205 | 251 | 200 | 160 | 275 |
| *Triacanthus biaculeatus* |  |  |  |  |  | 245 | 40 |  | 49 | 13 | 14 |  |
| *Tribolodon hakonensis* |  |  |  |  |  |  |  |  |  |  | 22 |  |
| *Upeneus japonicus* | 183 |  | 104 |  |  | 101 |  |  | 16 | 14 | 22 | 608 |

**Experiment 2**

(A) Glass Filter

| Species name | 0.5 L | | | | 2.0 L | | | |
| --- | --- | --- | --- | --- | --- | --- | --- | --- |
|  | 1 | 2 | 3 | 4 | 1 | 2 | 3 | 4 |
| Total read count | 49,169 | 48,896 | 49,398 | 49,598 | 49,000 | 48,690 | 49,232 | 48,618 |
| Total read count (after assignment to species) | 18,949 | 29,801 | 28,905 | 15,229 | 32,274 | 14,979 | 28,667 | 8,627 |
| *Abudefduf vaigiensis* | 0 | 0 | 0 | 0 | 0 | 0 | 4143 | 0 |
| *Acanthopagrus latus* | 0 | 0 | 0 | 0 | 0 | 0 | 0 | 590 |
| *Acanthopagrus schlegelii* | 0 | 0 | 0 | 0 | 1829 | 0 | 0 | 0 |
| *Bodianus reticulatus* | 0 | 0 | 0 | 0 | 4052 | 0 | 0 | 0 |
| *Chromis notata notata* | 0 | 0 | 0 | 0 | 0 | 0 | 0 | 0 |
| *Decapterus maruadsi* | 0 | 0 | 0 | 7218 | 1048 | 0 | 0 | 0 |
| *Ditrema sp.* | 6648 | 6236 | 9944 | 6125 | 13718 | 0 | 4884 | 2868 |
| *Engraulis japonicus* | 0 | 4444 | 6870 | 0 | 0 | 1361 | 0 | 0 |
| *Entomacrodus stellifer stellifer* | 0 | 0 | 0 | 0 | 0 | 0 | 5669 | 0 |
| *Hexagrammos otakii* | 0 | 0 | 0 | 0 | 0 | 0 | 0 | 0 |
| *Hexagrammos sp.* | 0 | 0 | 0 | 0 | 0 | 0 | 0 | 0 |
| *Hyporhamphus sajori* | 0 | 0 | 0 | 0 | 0 | 0 | 0 | 1018 |
| *Istigobius campbelli* | 0 | 0 | 0 | 0 | 0 | 0 | 0 | 0 |
| *Konosirus punctatus* | 0 | 0 | 0 | 0 | 0 | 0 | 0 | 0 |
| *Lateolabrax japonicus* | 0 | 0 | 0 | 0 | 0 | 0 | 0 | 0 |
| *Mugil cephalus* | 0 | 0 | 0 | 0 | 1895 | 0 | 2046 | 0 |
| *Oplegnathus fasciatus* | 0 | 0 | 0 | 0 | 0 | 0 | 0 | 491 |
| *Pagrus major* | 0 | 0 | 0 | 0 | 0 | 0 | 0 | 0 |
| *Parablennius yatabei* | 0 | 0 | 0 | 0 | 0 | 0 | 0 | 1453 |
| *Parajulis poecilepterus* | 0 | 0 | 0 | 0 | 0 | 0 | 0 | 0 |
| *Plotosus japonicus* | 0 | 0 | 0 | 0 | 0 | 0 | 0 | 0 |
| *Pseudolabrus sieboldi* | 0 | 0 | 0 | 0 | 0 | 0 | 0 | 0 |
| *Rudarius ercodes* | 0 | 3165 | 0 | 0 | 0 | 0 | 0 | 0 |
| *Sardinella zunasi* | 6804 | 7015 | 246 | 310 | 0 | 5918 | 3943 | 1056 |
| *Scomber sp.* | 0 | 0 | 4193 | 0 | 0 | 0 | 0 | 0 |
| *Scomberomorus niphonius* | 5497 | 0 | 0 | 0 | 1766 | 0 | 0 | 0 |
| *Sebastes sp.* | 0 | 0 | 0 | 0 | 0 | 0 | 2218 | 0 |
| *Seriola quinqueradiata* | 0 | 0 | 7652 | 1576 | 0 | 7700 | 0 | 0 |
| *Siganus fuscescens* | 0 | 2917 | 0 | 0 | 5089 | 0 | 3675 | 812 |
| *Stephanolepis cirrhifer* | 0 | 0 | 0 | 0 | 0 | 0 | 0 | 0 |
| *Takifugu sp.* | 0 | 6024 | 0 | 0 | 0 | 0 | 0 | 339 |
| *Thunnus sp.* | 0 | 0 | 0 | 0 | 0 | 0 | 0 | 0 |
| *Trachurus japonicus* | 0 | 0 | 0 | 0 | 2877 | 0 | 2089 | 0 |

(B) Sterivex

| Species name | 0.5 L | | | | 2.0 L | | | |
| --- | --- | --- | --- | --- | --- | --- | --- | --- |
|  | 1 | 2 | 3 | 4 | 1 | 2 | 3 | 4 |
| Total read count | 48,649 | 49,114 | 49,634 | 48,434 | 48,989 | 49,123 | 49,129 | 50,009 |
| Total read count (after assignment to species) | 22,974 | 27,767 | 21,855 | 29,434 | 30,715 | 25,946 | 19,050 | 22,974 |
| *Abudefduf vaigiensis* | 0 | 0 | 0 | 0 | 0 | 0 | 0 | 0 |
| *Acanthopagrus latus* | 0 | 0 | 0 | 0 | 0 | 0 | 0 | 0 |
| *Acanthopagrus schlegelii* | 0 | 0 | 0 | 0 | 0 | 0 | 3907 | 0 |
| *Bodianus reticulatus* | 0 | 0 | 0 | 0 | 2796 | 0 | 0 | 0 |
| *Chromis notata notata* | 0 | 0 | 0 | 0 | 0 | 0 | 0 | 0 |
| *Decapterus maruadsi* | 1913 | 2622 | 0 | 0 | 0 | 2356 | 0 | 1913 |
| *Ditrema sp.* | 5521 | 14733 | 12144 | 13958 | 13554 | 6335 | 2949 | 5521 |
| *Engraulis japonicus* | 0 | 0 | 0 | 1403 | 4148 | 3041 | 0 | 0 |
| *Entomacrodus stellifer stellifer* | 0 | 0 | 0 | 0 | 0 | 0 | 2915 | 0 |
| *Hexagrammos otakii* | 2992 | 0 | 0 | 0 | 0 | 0 | 0 | 2992 |
| *Hexagrammos sp.* | 0 | 0 | 0 | 0 | 0 | 0 | 0 | 0 |
| *Hyporhamphus sajori* | 3882 | 0 | 3901 | 0 | 0 | 0 | 0 | 3882 |
| *Istigobius campbelli* | 0 | 0 | 0 | 0 | 0 | 0 | 0 | 0 |
| *Konosirus punctatus* | 0 | 0 | 0 | 0 | 0 | 0 | 0 | 0 |
| *Lateolabrax japonicus* | 0 | 0 | 0 | 0 | 0 | 0 | 3290 | 0 |
| *Mugil cephalus* | 0 | 0 | 0 | 0 | 0 | 0 | 0 | 0 |
| *Oplegnathus fasciatus* | 3108 | 0 | 0 | 0 | 0 | 0 | 0 | 3108 |
| *Pagrus major* | 0 | 0 | 0 | 0 | 0 | 0 | 0 | 0 |
| *Parablennius yatabei* | 0 | 2933 | 0 | 0 | 1421 | 2999 | 0 | 0 |
| *Parajulis poecilepterus* | 0 | 0 | 0 | 2604 | 0 | 0 | 0 | 0 |
| *Plotosus japonicus* | 0 | 0 | 0 | 0 | 0 | 0 | 0 | 0 |
| *Pseudolabrus sieboldi* | 0 | 0 | 0 | 0 | 0 | 0 | 0 | 0 |
| *Rudarius ercodes* | 0 | 0 | 0 | 0 | 1496 | 0 | 0 | 0 |
| *Sardinella zunasi* | 118 | 3740 | 1028 | 1065 | 687 | 5139 | 2671 | 118 |
| *Scomber sp.* | 0 | 0 | 0 | 0 | 0 | 0 | 0 | 0 |
| *Scomberomorus niphonius* | 0 | 0 | 0 | 0 | 0 | 0 | 0 | 0 |
| *Sebastes sp.* | 0 | 0 | 0 | 0 | 0 | 0 | 0 | 0 |
| *Seriola quinqueradiata* | 0 | 0 | 0 | 1798 | 898 | 2927 | 3318 | 0 |
| *Siganus fuscescens* | 0 | 3739 | 4782 | 4690 | 5715 | 3149 | 0 | 0 |
| *Stephanolepis cirrhifer* | 0 | 0 | 0 | 0 | 0 | 0 | 0 | 0 |
| *Takifugu sp.* | 2329 | 0 | 0 | 3916 | 0 | 0 | 0 | 2329 |
| *Thunnus sp.* | 0 | 0 | 0 | 0 | 0 | 0 | 0 | 0 |
| *Trachurus japonicus* | 3111 | 0 | 0 | 0 | 0 | 0 | 0 | 3111 |

(C) QuickConc Vacuum

| Species name | 0.5 L | | | | 2.0 L | | | |
| --- | --- | --- | --- | --- | --- | --- | --- | --- |
|  | 1 | 2 | 3 | 4 | 1 | 2 | 3 | 4 |
| Total read count | 49,411 | 49,088 | 49,531 | 49,438 | 49,547 | 48,812 | 49,285 | 49,032 |
| Total read count (after assignment to species) | 22,665 | 31,340 | 18,221 | 23,244 | 29,258 | 31,056 | 31,009 | 31,943 |
| *Abudefduf vaigiensis* | 0 | 0 | 0 | 0 | 0 | 0 | 0 | 0 |
| *Acanthopagrus latus* | 0 | 0 | 0 | 0 | 0 | 0 | 984 | 0 |
| *Acanthopagrus schlegelii* | 2837 | 3849 | 2053 | 0 | 1774 | 346 | 1941 | 0 |
| *Bodianus reticulatus* | 0 | 0 | 0 | 0 | 1363 | 553 | 1034 | 434 |
| *Chromis notata notata* | 0 | 0 | 0 | 88 | 0 | 0 | 0 | 0 |
| *Decapterus maruadsi* | 0 | 0 | 0 | 0 | 284 | 1849 | 0 | 993 |
| *Ditrema sp.* | 8772 | 4985 | 2386 | 20967 | 8105 | 9082 | 6587 | 18523 |
| *Engraulis japonicus* | 0 | 0 | 2269 | 0 | 851 | 0 | 2063 | 1843 |
| *Entomacrodus stellifer stellifer* | 0 | 3235 | 0 | 0 | 869 | 0 | 0 | 288 |
| *Hexagrammos otakii* | 0 | 3684 | 0 | 0 | 577 | 0 | 0 | 0 |
| *Hexagrammos sp.* | 0 | 0 | 0 | 0 | 829 | 0 | 0 | 0 |
| *Hyporhamphus sajori* | 0 | 0 | 7777 | 0 | 1297 | 1550 | 696 | 135 |
| *Istigobius campbelli* | 0 | 0 | 56 | 0 | 0 | 0 | 0 | 0 |
| *Konosirus punctatus* | 0 | 0 | 0 | 0 | 1561 | 0 | 0 | 0 |
| *Lateolabrax japonicus* | 0 | 0 | 0 | 0 | 0 | 1455 | 0 | 0 |
| *Mugil cephalus* | 2398 | 0 | 0 | 0 | 0 | 356 | 0 | 434 |
| *Oplegnathus fasciatus* | 0 | 1138 | 0 | 0 | 761 | 1515 | 0 | 407 |
| *Pagrus major* | 0 | 0 | 0 | 0 | 959 | 0 | 0 | 0 |
| *Parablennius yatabei* | 0 | 0 | 0 | 2189 | 0 | 3054 | 0 | 421 |
| *Parajulis poecilepterus* | 0 | 0 | 0 | 0 | 815 | 0 | 0 | 0 |
| *Plotosus japonicus* | 0 | 0 | 0 | 0 | 0 | 19 | 0 | 0 |
| *Pseudolabrus sieboldi* | 0 | 0 | 0 | 0 | 0 | 0 | 0 | 86 |
| *Rudarius ercodes* | 0 | 0 | 0 | 0 | 0 | 0 | 0 | 0 |
| *Sardinella zunasi* | 3247 | 0 | 3680 | 0 | 2230 | 3031 | 2095 | 615 |
| *Scomber sp.* | 0 | 0 | 0 | 0 | 0 | 0 | 0 | 0 |
| *Scomberomorus niphonius* | 0 | 0 | 0 | 0 | 0 | 703 | 489 | 700 |
| *Sebastes sp.* | 0 | 0 | 0 | 0 | 1314 | 2053 | 0 | 0 |
| *Seriola quinqueradiata* | 0 | 1141 | 0 | 0 | 1231 | 1449 | 0 | 686 |
| *Siganus fuscescens* | 5411 | 3771 | 0 | 0 | 3943 | 2656 | 13141 | 5768 |
| *Stephanolepis cirrhifer* | 0 | 0 | 0 | 0 | 0 | 0 | 1979 | 0 |
| *Takifugu sp.* | 0 | 6337 | 0 | 0 | 0 | 478 | 0 | 0 |
| *Thunnus sp.* | 0 | 3200 | 0 | 0 | 0 | 0 | 0 | 610 |
| *Trachurus japonicus* | 0 | 0 | 0 | 0 | 495 | 907 | 0 | 0 |

Table S6. Detailed list of fish species in aquarium tank

| Tank A_ID | Species name | Tank B_ID | Species name |
| --- | --- | --- | --- |
| 1 | *Gymnomuraena zebra* | 1 | *Gymnothorax kidako* |
| 2 | *Antennarius commersoni* | 2 | *Gymnothorax reticularis* |
| 3 | *Echeneis naucrates* | 3 | *Epinephelus akaara* |
| 4 | *Caesio cuning* | 4 | *Epinephelus areolatus* |
| 5 | *Pterocaesio digramma* | 5 | *Epinephelus awoara* |
| 6 | *Pseudanthias pleurotaenia* | 6 | *Epinephelus septemfasciatus* |
| 7 | *Pseudanthias squamipinnis* | 7 | *Epinephelus fasciatus* |
| 8 | *Epinephelus coioides* | 8 | *Goniistius zonatus* |
| 9 | *Paracirrhites forsteri* | 9 | *Oplegnathus fasciatus* |
| 10 | *Chromis viridis* | 10 | *Cirrhitichthys aureus* |
| 11 | *Dascyllus melanurus* | 11 | *Microcanthus strigatus* |
| 12 | *Dascyllus trimaculatus* | 12 | *Semicossyphus reticulatus* |
| 13 | *Chrysiptera cyanea* | 13 | *Stephanolepis cirrhifer* |
| 14 | *Chrysiptera parasema* | 14 | *Plotosus japonicus* |
| 15 | *Mulloidichthys　vanicolensis* | 15 | *Girella punctata* |
| 16 | *Parupeneus ciliatus* | 16 | *Girella melanichthys* |
| 17 | *Chaetodon auripes* | 17 | *Gymnothorax pseudothyrsoideus* |
| 18 | *Chaetodon ephippium* | 18 | *Scorpaena miostoma* |
| 19 | *Chaetodon melannotus* | 19 | *Pterois lunulata* |
| 20 | *Hemitaurichthys polylepis* | 20 | *Oplegnathus punctatus* |
| 21 | *Genicanthus watanabei* | 21 | *Plectroglyphidodon altus* |
| 22 | *Pomacanthus sexstriatus* | 22 | *Inimicus japonicus* |
| 23 | *Bolbometopon bicolor* | 23 | *Plectorhinchus cinctus* |
| 24 | *Scarus ghobban* | 24 | *Pagrus major* |
| 25 | *Naso lituratus* | 25 | *Acanthopagrus schlegelii* |
| 26 | *Acanthurus olivaceus* | 26 | *Rhabdosargus sarba* |
| 27 | *Odonus niger* | 27 | *Lateolabrax japonicus* |
| 28 | *Aluterus scriptus* | 28 | *Epinephelus morrhua* |
| 29 | *Ostracion rhinorhynchos* | 29 | *Epinephelus septemfasciatus* |
| 30 | *Chilomycterus reticulatus* | 30 | *Goniistius quadricornis* |
| 31 | *Alectis ciliaris* | 31 | *Goniistius zebra* |
| 32 | *Pseudanthias rubrizonatus* | 32 | *Gymnothorax ypsilon* |
| 33 | *Paracanthurus hepatus* | 33 | *Gymnothorax reticularis* |
| 34 | *Parapercis polyophtalma* | 34 | *Triakis scyllium* |
| 35 | *Coris aygula* | 35 | *Heterodontus japonicus* |
| 36 | *Chaetodon kleinii* | 36 | *Epinephelus bruneus* |
| 37 | *Chaetodon lunula* | 37 | *Sebastiscus marmoratus* |
| 38 | *Centropyge bicolor* | 38 | *Cirrhitichthys aureus* |
| 39 | *Acanthurus japonicus* |  |  |
| 40 | *Siganus virgatus* |  |  |
| 41 | *Arothron hispidus* |  |  |
| 42 | *Parupeneus multifasciatus* |  |  |
| 43 | *Coris gaimard* |  |  |
| 44 | *Acanthurus pyroferus* |  |  |
| 45 | *Decapterus maruadsi* |  |  |
| 46 | *Diodon holocanthus* |  |  |
| 47 | *Diodon hystrix* |  |  |
| 48 | *Ditrema temminckii temminckii* |  |  |
| 49 | *Enophrys diceraus* |  |  |
| 50 | *Epinephelus trimaculatus* |  |  |
| 51 | *Halichoeres tenuispinis* |  |  |
| 52 | *Naso unicornis* |  |  |
| 53 | *Omobranchus elegans* |  |  |
| 54 | *Paramonacanthus japonicus* |  |  |
| 55 | *Parapristipoma trilineatum* |  |  |
| 56 | *Platax teira* |  |  |
| 57 | *Pomacanthus xanthometopon* |  |  |
| 58 | *Siganus virgatus* |  |  |
| 59 | *Syngnathus schlegeli* |  |  |

| Tank C_ID | Species name | Tank D_ID | Species name |
| --- | --- | --- | --- |
| 1 | *Heterodontus japonicus* | 1 | *Mugil cephalus* |
| 2 | *Hemitriakis japonica* | 2 | *Paracentropogon rubripinnis* |
| 3 | *Mustelus manazo* | 3 | *Sillago japonica* |
| 4 | *Triakis scyllium* | 4 | *Nuchequula nuchalis* |
| 5 | *Carcharhinus plumbeus* | 5 | *Ostorhinchus notatus* |
| 6 | *Carcharhinus brachyurus* | 6 | *Upeneus japonicus* |
| 7 | *Carcharhinus obscurus* | 7 | *Ditrema temmincki temmincki* |
| 8 | *Carcharhinus brevipinna* | 8 | *Parachirus sp.* |
| 9 | *Carcharhinus limbatus* | 9 | *Dictyosoma burgeri* |
| 10 | *Sphyrna lewini* | 10 | *Petroscirtes breviceps* |
| 11 | *Dasyatis matsubarai* | 11 | *Repomucenus richardsonii* |
| 12 | *Taeniura meyeni* | 12 | *Acentrogobius pflaumii* |
| 13 | *Gymnura japonica* | 13 | *Triacanthus biaculeatus* |
| 14 | *Myliobatis tobijei* | 14 | *Pholis nebulosa* |
| 15 | *Aetobatus narinari* | 15 | *Rudarius ercodes* |
| 16 | *Gymnothorax kidako* | 16 | *Thamnaconus modestus* |
| 17 | *Sardinops melanostictus* | 17 | *Gerres oyena* |
| 18 | *Coryphaena hippurus* | 18 | *Apogon niger* |
| 19 | *Echeneis naucrates* | 19 | *Scorpaenodes littoralis* |
| 20 | *Kaiwarinus equula* | 20 | *Istigobius campbelli* |
| 21 | *Trachurus japonicus* | 21 | *Suezichthys gracilis* |
| 22 | *Pseudocaranx dentex* | 22 | *Cirrhilabrus temminckii* |
| 23 | *Hapalogenys sennin* | 23 | *Girella melanichthys* |
| 24 | *Pagrus major* |  |  |
| 25 | *Gymnocranius griseus* |  |  |
| 26 | *Lethrinus genivittatus* |  |  |
| 27 | *Lethrinus nebulosus* |  |  |
| 28 | *Labracoglossa argenteiventris* |  |  |
| 29 | *Rhynchopelates oxyrhynchus* |  |  |
| 30 | *Semicossyphus reticulatus* |  |  |
| 31 | *Prionurus scalprum* |  |  |
| 32 | *Sphyraena pinguis* |  |  |
| 33 | *Scomber japonicus* |  |  |
| 34 | *Takifugu xanthopterus* |  |  |
| 35 | *Lagocephalus sceleratus* |  |  |

Figure S1. eDNA metabarcoding species accumulation curves. Species accumulation curves for Experiment 1 (A) and Experiment 2 (B) show how many species on average are detected with increasing number of reads across three concentration methods: Glass filter (blue), Sterivex (green), and QuickConc Vacuum (red).

Figure S2. Sample-size-based rarefaction and extrapolation curves for fish taxon richness estimated using iNEXT. Curves for Experiment 1 (A) and Experiment 2 (B) are shown separately for glass fiber filter (GF), Sterivex, and QuickConc Vacuum (QCV) at each water volume. GF, Sterivex, and QCV are shown in light blue, light green, and light coral, respectively. For each method-volume condition, unrarefied post-quality-control metabarcoding read counts from four biological replicates were pooled by taxon before analysis. Because read counts from the four biological replicates were pooled before iNEXT analysis, the observed points represent condition-level pooled taxon richness and are not directly equivalent to replicate-level detected-species richness. Solid lines denote rarefaction, dashed lines denote extrapolation to twice the observed read count, points indicate observed richness at the reference sample size, and shaded bands with boundary lines represent 95% confidence intervals based on 50 bootstrap replications. Only Hill number q = 0 (taxon richness) was analyzed. Read counts were used as sequencing observations to evaluate sequencing-depth-dependent taxon accumulation and sampling completeness, not as estimates of organismal abundance. Because the confidence intervals were narrow, the shaded bands and their boundary lines overlap the richness curves in several panels.
